# Spatial Transcriptomics Reveals a T Cell‐Mediated Microglial Activation Axis of Immune Checkpoint Inhibition‐Related Adverse Events in the Brain

**DOI:** 10.1002/advs.77733

**Published:** 2026-09-30

**Authors:** Devyani Swami, Sanad M. El‐Khatib, Suhas Sureshchandra, Elise Nguyen, Janaki Manoja Vinnakota, Robert Zeiser, Shivashankar Othy, Munjal M. Acharya

**Affiliations:** ^1^ Department of Anatomy & Neurobiology, School of Medicine University of California Irvine USA; ^2^ Department of Physiology & Biophysics School of Medicine University of California Irvine USA; ^3^ Department of Hematology and Oncology Freiburg University Medical Center Germany; ^4^ Department of Radiation Oncology School of Medicine University of California Irvine USA

**Keywords:** anti‐CTLA‐4, anti‐PD‐1, astrocytes, immune checkpoint inhibition, irAEs, microglia, neuroinflammation, spatial transcriptomics, synapse, T cells

## Abstract

Immune checkpoint inhibitors (ICIs) targeting CTLA‐4 and PD‐1 have transformed cancer therapy, yet their immune‐related adverse events (irAEs) in various tissues, including the brain, remain poorly defined. ICI perturbs hippocampal‐dependent memory function by derailing neuro‐immune homeostasis and compromising synaptic integrity. However, the cellular and molecular mechanisms by which ICI alters regional interactions are not well understood. Using bulk RNA‐sequencing and MERFISH spatial transcriptomics in a syngeneic murine melanoma model, we show that ICI treatment remodels hippocampal gene expression and cellular organization. While tumor burden itself altered brain physiology, ICI distinctly modified the abundance and transcriptional states of neurons, microglia, astrocytes, oligodendrocytes, and T cells, with coordinated induction of inflammatory, synaptic, and immune‐response programs. Complementary analysis of human postmortem brain samples confirmed microglial activation as a key hallmark of ICI‐treated brains, highlighting the translational relevance of our findings. Finally, using a conditional deletion model, we show that T cells are indispensable for ICI‐driven microglial activation. Altogether, our results identify a T cell–microglia crosstalk axis as a driving mechanism of the pathophysiology of irAEs in the brain and provide a high‐resolution spatial model for better understanding neuroinflammatory responses during ICI‐induced unleashing of anti‐tumor immunity.

## Graphical abstract

Immune checkpoint inhibition (ICI) reshapes the hippocampal neuroimmune niche. Spatial transcriptomic profiling and human brain histological analyses identify neuroinflammation and altered neuronal and glial programs. Notably, T cell depletion prevents ICI‐driven microglial activation, establishing the T cell–microglia crosstalk axis as a driving mechanism of the pathophysiology of immune‐related adverse events in the brain.

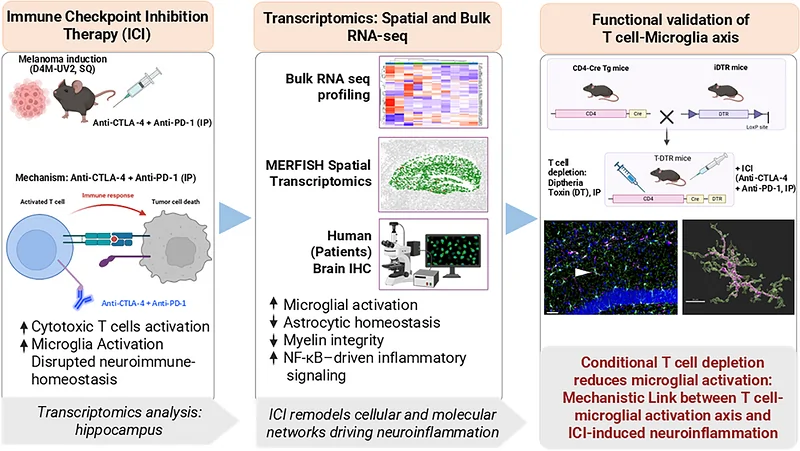

## 1 Introduction

Immune checkpoint inhibitors (ICIs), targeting programmed cell death protein 1 (PD‐1) and cytotoxic T‐lymphocyte‐associated protein 4 (CTLA‐4), are transformative breakthroughs in modern cancer care. ICIs improve cancer treatment by restoring anti‐tumor immunity, leading to major improvements in patient survival and overall quality of life [1]. ICI therapies continue to expand rapidly and are being translated across a wide range of advanced or metastatic cancers, including lung, renal, gastrointestinal, breast, and prostate cancers [2, 3]. ICIs are administered either alone or in combination regimens to reinvigorate exhausted immune cells, particularly cytotoxic (CD8^+^) T cells, that play a central role in eliminating tumors [4, 5]. However, despite life‐saving therapeutic benefit, a potential risk associated with ICIs is off‐target effects and immune‐related toxicities of normal tissues, including the central nervous system (CNS) [6, 7]. Neurocognitive and neuroinflammatory sequelae of ICI therapy and relevant mechanisms are not well understood.

Emerging clinical evidence suggests that ICI contributes to cognitive decline and other neurological complications. Findings from small clinical studies showed one case of grade 3 cognitive dysfunction following anti‐PD‐1 treatment [8] and seven out of 18 survivors showed subjective cognitive impairment 5.6 years post‐anti‐CTLA‐4 therapy [9]. More recently, up to a 26% incidence of clinically significant cancer‐related cognitive impairment was observed in ICI‐treated patients across lung cancer, GI cancers, and melanoma [10]. Additionally, AILEEN study, including a large cohort of subjects (*N* = 107), demonstrated that melanoma survivors treated with ICIs had notable cognitive and psychosocial difficulties, with 34% showing objective cognitive impairment and 36% reporting clinically meaningful cognitive problems two years post‐treatment [11]. Combination ICI‐treated patients also experienced fatigue, deficits in verbal memory, processing speed, and executive function. To what extent these neurocognitive effects are driven by cancer‐associated changes in brain physiology versus cancer treatment with ICI is a major question. Because clinical applications of ICIs are rapidly expanding, uncovering the cellular and molecular bases of potential detrimental effects on brain function is important to improve the quality of life of survivors.

ICI‐related immune‐mediated toxicities, collectively termed immune‐related adverse events (irAEs), can occur in various off‐target tissues, including the nervous system (central as well as peripheral). The clinical manifestations of these irAEs are diverse and often include encephalitis, headache, seizures, fatigue, myasthenia gravis, Guillain–Barré syndrome, and peripheral neuropathy [12, 13]. Clinical reports indicate that most irAEs emerge early after treatment initiation, with 65.5% of cases (19 of 29) developing within approximately 15 weeks. In a single‐center longitudinal study of 25 patients treated with pembrolizumab, 32% developed treatment‐related neurocognitive impairment. Similarly, a cross‐sectional study reported functional deficits in working memory and verbal memory in up to 41% of patients treated with ipilimumab [14]. In addition, ICI‐related neurotoxicity, particularly following anti‐CTLA‐4 therapy, has been linked to demyelination and CNS autoimmunity, with myelin‐reactive CD4^+^ T cells identified in the brains of affected patients [15]. Despite these growing clinical reports, the precise mechanisms underlying ICI‐associated neuroinflammatory, neurodegenerative, and neurocognitive complications remain poorly understood [14]. Thus, ICI‐associated neurological toxicity represents an underrecognized and clinically meaningful complication that warrants further investigation.

Preclinical studies are establishing new paradigms for how unleashing anti‐tumor immunity with ICI therapies affects brain function. In a murine model of ICI‐responsive melanoma, combinatorial ICI treatment (anti‐PD‐1 + anti‐CTLA‐4; Combi‐ICI) effectively controlled tumor growth but was accompanied by marked impairments in hippocampus‐dependent learning and memory, and higher‐order memory consolidation processes [16]. Combi‐ICI alone (without tumor) was sufficient to negatively affect brain function. While neurogenesis and neuronal plasticity were unaltered, cognitive decline following combi‐ICI was associated with glial dysfunction characterized by microglial activation and astrogliosis, and amplified neuroinflammation, which disrupted synaptic and neuronal integrity. Combi‐ICI expanded the brain pools of T cells, upregulated MHC‐II on myeloid cells, and exaggerated severity of CNS autoimmunity [16]. Another study demonstrated that anti‐PD‐1 therapy‐modulated T cells destabilize the blood–brain barrier in a Dickkopf‐related protein 1 (DKK1) dependent manner [17]. Blocking PD1 (in the absence of cancer) also induces neurocognitive deficits in mice and is associated with microglial morphology changes and upregulation of MHC‐II [18]. Collectively, these studies have established microglia and their aberrant activation during ICI as a key hallmark of irAEs in the brain. Correspondingly, postmortem analyses of brain tissue from patients treated with anti‐PD‐1 therapy verified microglial activation [18]. In addition, an earlier murine study showed that BALB/c mice bearing colorectal carcinoma and treated with combined radiation and immunotherapy exhibited impaired object recognition and increased anxiety‐like behavior, indicative of hippocampus‐ and amygdala‐dependent dysfunction [19]. Overall, findings in preclinical murine models suggest that ICI therapy can perturb brain function by altering immune regulatory networks among resident cell populations. However, how ICI‐induced changes in glia and their immune states are spatially organized to impair hippocampal‐dependent learning, memory, and cognitive processing are not well defined.

Understanding the organizational landscape of irAEs is essential for defining how ICI therapy disrupts brain function and for guiding the development of targeted strategies that protect the most affected cell populations while preserving the life‐saving anti‐tumor efficacy. Thus, our current study establishes a spatial framework of cellular interactions, immune pathways, and the role of T cells in orchestrating neuroimmunological responses during ICI therapy. Using a combination of high‐resolution targeted spatial transcriptomics, immunohistochemistry, and genetic cellular depletion approaches, we define how ICI distinctly alters hippocampal cellular composition and gene expression patterns, highlighting pathophysiological crosstalk among neurons, glia, and immune cells.

## 2 Materials and Methods

### 2.1 Animal Model and Treatments

All mice used in this study were housed and cared for in accordance with National Institutes of Health (NIH) guidelines and protocols (protocol numbers: AUP‐24‐131 and AUP‐25‐135) approved by the Institutional Animal Care and Use Committee (IACUC). Adult male wild‐type (WT) mice aged 10–12 weeks were randomly assigned to one of the following experimental groups: (i) WT mice receiving isotype‐matched control antibodies (intraperitoneal, IP); (ii) WT mice bearing D4M‐3A.UV2 tumors and treated with isotype‐matched control antibodies (IP); (iii) WT mice treated with immune checkpoint inhibitors (ICIs), including anti–CTLA‐4 (1 mg per dose, three times weekly, IP) and anti‐PD‐1 (200 µg per dose, twice weekly, IP), Combi‐ICI; and (iv) WT mice bearing D4M‐3A.UV2 tumors were treated with the same combination ICI regimen for three weeks. The Combi‐ICI treatment was given every other day (thrice weekly for anti‐CTLA‐4 and twice weekly for anti‐PD‐1).

Tumor induction and Combi‐ICI treatment essentially followed the same experimental protocol as described previously [16]. For tumor induction, mice in the cancer groups received bilateral intradermal injections of 5 × 10^5^ syngeneic D4M‐3A.UV2 melanoma cells. Tumors were allowed to engraft for one week prior to initiating ITC or ICI treatments. Our previous study confirmed that D4M‐3A.UV2 do not spontaneously metastasize from the site of injection to the CNS [16]. Tumor growth through vertical and horizontal axes was measured using electronic calipers, and tumor volume was calculated as 0.5 × *a* × *b* ^2^ (*a*, minimum diameter, and *b*, maximum diameter). Mice were treated with Combi‐ICI (as above) when the tumor volumes reached 20–30 mm^3^ around 7 days post‐tumor induction. Following completion of the ICI treatment regimen, mice were euthanized 72 h after the final injection, and brains were harvested for downstream analyses.

### 2.2 Tissue Preparation, Transcriptomics, and Data Processing

For bulk RNA‐seq data, fresh‐frozen hippocampi from *N* = 4 brains per group were excised and sent to Azenta Life Science. Briefly, total RNA extraction, library preparation, and sequencing were performed; RNA samples were quantified using a Qubit 2.0 Fluorometer (Life Technologies, Carlsbad, CA, USA), and RNA integrity was checked using an Agilent TapeStation 4200 (Agilent Technologies, Palo Alto, CA, USA). We sequenced the libraries on the Illumina NovaSeq X Plus instrument. The samples were sequenced using a 2 × 150 bp paired‐end (PE) configuration, targeting approximately 30 M reads/sample. Further details are available in Supporting Data. For differential expression gene analysis (DEGs) files, read counts were normalized to adjust for various factors, such as variations in sequencing yield between samples (software package DESeq2 v.1.16.1). For analysis, A Wald test was used to test for changes in gene expression (software package DESeq2, v.1.16.1). Genes with adjusted *p*‐values < 0.05 and an absolute log2‐fold change > 1 were considered statistically significant differentially expressed genes (DEGs). For subsequent DEG analyses, Benjamini–Hochberg adjusted *P*‐values and statistical *P*‐values < 0.001 were used for comparative analysis across datasets.

For Spatial transcriptomics using MERFISH, whole brains were carefully harvested from mice following decapitation and immediately placed into tissue‐embedding plastic molds. The prechilled OCT was poured onto the tissue‐embedding plastic mold and later transferred into isopentane and a liquid nitrogen bath until the OCT completely solidified and turned white. To ensure RNA integrity, at least the first 50 µm of tissue sections were discarded prior to collecting evaluable samples. Subsequently, 10 µm sections from the OCT‐embedded tissue blocks were mounted onto slides and processed for probe hybridization and gel embedding according to the MERFISH protocol. The custom 300‐gene MERFISH panel (see Table S1) encompassed neuro‐inflammatory, glial, neuronal, T and B cell gene sets with the goal of defining neuro‐immune function in melanoma ICI treatment.

Prepared slides were assembled into the MERSCOPE Flow Chamber and loaded into the MERSCOPE instrument along with the appropriate imaging cartridge. Raw images were processed using the MERSCOPE software pipeline to generate spatial genomics output files ready for downstream analysis. These outputs included detected transcripts and their three‐dimensional spatial coordinates, provided as cell metadata, experiment metadata (JSON), cell boundary files (HDF5), and MERSCOPE Visualizer files (VZG). The transcript‐level output contained the identities and three‐dimensional spatial coordinates (*x*, *y*, and *z*) of detected transcripts, whereas the cell metadata contained the two‐dimensional centroid coordinates (center_*x* and center_*y*) of individual segmented cells. The center_*x* and center_*y* coordinates were used for downstream spatial mapping and proximity analyses.

Using the MERSCOPE Visualizer software, AnnData (HDF5) files were loaded, and hippocampal ROIs were selected Figure S1A. Using the MERSCOPE Visualizer software, AnnData (HDF5) files were loaded, and hippocampal ROIs were selected. Briefly, Initial MERFISH preprocessing was performed from MERSCOPE‐exported HDF5 files using Scanpy. HDF5 files were loaded as AnnData objects, and genes containing “Blank” in their names were removed before downstream analysis. Cells were restricted to predefined hippocampal regions of interest based on MERSCOPE custom cell‐group annotations. Cell‐level quality filtering was performed using total expression per cell, and cells with total expression ≤25 were excluded. Volume‐based filtering was evaluated but was not applied in the final workflow. The same filtering criteria were applied across all datasets before integration. After filtering, expression matrices were normalized using Scanpy total‐count normalization followed by log1p transformation. Data were scaled with a maximum value of 10, and PCA was performed using the ARPACK solver. A nearest‐neighbor graph was constructed using 10 neighbors and the first 20 principal components, followed by UMAP visualization and initial Leiden clustering. Cluster‐level signatures were generated by calculating the mean expression of each gene within each cluster. For each experimental condition, cells were grouped by biological replicate and Leiden cluster. The cell counts and proportional cluster representation were calculated from each replicate. Replicate‐level cluster stability results are provided in Table S2. Processed AnnData objects were saved as HDF5 files and converted to Seurat RDS format for downstream integration, clustering, annotation, and differential expression analysis.

### 2.3 Analysis of MERFISH Spatial Transcripts

Cell type markers were based on the Gene panel‐specific MERSCOPE Codebooks and were customized using MERSCOPE Gene Panel Design Software. A cell‐by‐gene matrix was imported from MERSCOPE visualizer software as an HDF5 file and was loaded into Seurat for downstream analysis. The merged MERFISH AnnData object was normalized using total‐count normalization with a target sum of 10 000 counts per cell, followed by log1p transformation. Highly variable genes were selected using the Seurat‐flavor method with *n*_top_genes = 300, and the data were scaled with max_value = 10 before PCA.

Cells were first integrated across datasets using Seurat, followed by dimensionality reduction and graph‐based clustering. For final dataset integration, condition‐specific Seurat objects corresponding to Control, Control ICI, Melanoma, and Melanoma ICI groups were loaded independently and assigned dataset identities. The original expression assay was used for preprocessing. Variable features were identified within each dataset using Seurat's FindVariableFeatures function with the vst selection method. Because the MERFISH assay was based on a targeted 300‐gene panel focused on neuroinflammation and glial transcriptional programs, integration features were selected using SelectIntegrationFeatures with nfeatures = 300. Each dataset was scaled and subjected to PCA before integration. Dataset‐level integration and batch correction were performed using Seurat reciprocal PCA‐based anchor integration, with integration anchors identified using dimensions 1–20. Following integration with IntegrateData, the integrated assay was scaled, and PCA was rerun using 20 principal components. A shared nearest‐neighbor graph was constructed using PCs 1–20, graph‐based clustering was performed at resolution 1, and UMAP visualization was generated from the PCA reduction using dimensions 1–20. Clustering was evaluated across resolutions 0.2, 0.4, 0.6, 0.8, 1, and 1.2. Resolution 1 was selected because it preserved major canonical cell‐type separation while avoiding excessive cluster fragmentation. The final integrated object was saved as MERFISH_integrated_final_res1.0.rds and used for downstream visualization and annotation.

For each Leiden cluster, cluster‐specific marker genes were identified (imported as a gene panel). Cell‐type identities were assigned using a marker overlap‐based scoring approach. Cluster‐specific marker genes were identified from the integrated MERFISH Seurat object using the original expression assay. Cluster identities were set to Seurat clusters, and positive cluster markers were identified using Seurat FindAllMarkers with only.pos = TRUE, min.pct = 0.1, and logfc.threshold = 0.25. Cell‐type annotation was performed using a marker‐overlap scoring approach. Marker‐overlap score = number of cluster marker genes overlapping with the curated marker list for each cell type. Curated marker gene sets were defined for astrocytes, microglia, oligodendrocytes, excitatory neurons, GABAergic neurons, cholinergic neurons, T cells, B cells, complement‐enriched populations, and synaptic‐function–enriched populations. All marker genes were converted to uppercase before comparison. For each cluster, the marker‐overlap score was calculated as the number of cluster marker genes overlapping with the curated marker list for each cell type. Each cluster was assigned to the cell type with the highest marker‐overlap score, and the number of matched genes was retained as an annotation‐confidence measure. Clusters expressing ≥2 T‐cell markers were annotated as T cells, clusters expressing ≥2 complement pathway genes were labeled as complement‐enriched, and clusters meeting both criteria were annotated as T cells/complement‐enriched. Clusters with low or mixed marker‐overlap scores were manually reviewed using canonical marker expression and cluster‐specific markers and were retained as enriched or mixed populations when appropriate. Cell‐type labels were stored as metadata and used for visualization and downstream comparison without altering the underlying clustering structure.

Leiden clusters within each cell type were relabeled with simplified identifiers (e.g., A1–An for astrocytes, O1–On for oligodendrocytes, T1–Tn for T cells, and M1–Mn for microglia), without altering the underlying clustering structure. These relabeled cluster identities were stored as metadata and used exclusively for visualization. To assess condition‐specific distributions of cell type–specific clusters, UMAP embeddings were visualized using DimPlot and split by experimental condition. Importantly, the same UMAP coordinate system was used across all split panels, enabling direct comparison of cluster abundance and spatial organization across conditions. A volcano plot across the cell types and across the dataset was generated using the EnhancedVolcano package, with log_2_ fold change (avg_log2FC) plotted against adjusted *p*‐value (−log_10_ scale). Upregulated and downregulated genes were visualized simultaneously, enabling direct comparison of transcriptional changes between conditions. Overlapping gene sets exhibiting opposite regulation between groups were identified using set intersection analysis.

### 2.4 Gene Ontology

Functional enrichment analysis of DEGs for selected cell types was performed using Metascape. Enriched gene ontology (GO) biological processes and pathway terms were identified using default Metascape parameters. Bubble plots were generated in R using ggplot2 to visualize selected enriched GO terms across experimental conditions. Enrichment patterns were compared across experimental groups (Control, Control + ICI, Melanoma, and Melanoma + ICI) and microglial subclusters. Bubble color represents the scaled average expression of genes associated with each term, while bubble size indicates the percentage of cells expressing the corresponding genes within each cluster.

### 2.5 Dual‐Immunofluorescence Staining, Confocal Microscopy, and 3D Algorithm‐Based Volumetric Quantification

#### 2.5.1 Mouse Brains

Free‐floating mouse brain sections (*N* = 4–8 brains/group, 2–3 sections/ brain) with visible hippocampi were chosen for immunohistochemistry (IHC). Primary antibodies to characterize microglial activation include rat anti‐mouse CD68 (1:500, Bio‐Rad, Cat# MCA1957; RRID: AB_322219), and rabbit anti‐IBA1 (1:500, Wako, Cat# 019–19741; RRID: AB_839504).Secondary antibodies used for CD68 marker 1:1000; Goat anti Rat AF 647 (Abcam; Cat. ab150159), and 1:500; Goat anti Rabbit AF 488 (FisherSci; Cat. a11008) for IBA1 marker]

For staining, tissue sections were first washed 3 times in phosphate‐buffered saline (1 × PBS, 100 mm, pH 7.4, FisherSci) and, when necessary, antigen retrieval was performed using citrate buffer incubation (10 mm, pH 6 with 0.05% Tween‐20, Sigma) at 70°C for 45 min. Tissues were then incubated in borate buffer (pH 8.5, 100 mm, Sigma, Cat. B0394) for 10 min, followed by three 5‐min washes in 1 × PBS. Tissues were then blocked in serum (10% normal goat or donkey, NGS or NDS) in PBS with 0.01% Triton X‐100 (NDS, Jackson ImmunoResearch Labs Cat# 017‐000‐121, RRID: AB_2337258; NGS, Jackson ImmunoResearch Labs Cat# 005‐000‐121, RRID: AB_2336990). Following an hour of incubation in the blocking solution, the tissues were incubated in primary antibodies. Primary antibodies were prepared in 0.01% Triton X‐100 (Sigma), 3% NGS or NDS in PBS, and incubated for at least 12 h at 4°C in a shaker incubator (Enviro‐Genie Scientific). The tissues were washed in 1x PBS (3 times) and then incubated with the secondary antibodies for 1 h. Finally, sections were counterstained with DAPI nuclear dye in PBS for 15 min (1 µmol/L, FisherSci, Cat# D1306, RRID: AB_2629482). Finally, sections were washed in PBS and then mounted on the Superfrost slides (FisherSci) using Antifade mounting medium (VectaShield Cat# H‐1000‐10). For immunofluorescence analysis, each tissue section was treated as an independent data point for quantification.

#### 2.5.2 Human Brain Immunofluorescence Staining

FFPE sections from human cortical tissues (frontal cortex) isolated from ICI‐treated or non‐tumor control brains post‐mortem were gifts from the Department of Nuclear Medicine, LMU Munich, Medical Center—University of Freiburg, and approved by the ethics committee of the University of Freiburg (protocol number 340/19). The Institutional Biosafety Committee (IBC) of the University of California, Irvine approved the use of human brain tissue samples for this study (protocol number BUA‐R218). Briefly, the human brain tissue samples from ICI‐treated cases and non‐ICI control cases were obtained as listed in Table S3. Briefly, all patients in the ICI‐treated group were male, with a median age of 65 years. Treatments included pembrolizumab, nivolumab, or combined ipilimumab and nivolumab, and the underlying malignancies included lung cancer, melanoma, and head and neck cancer. The control group included two males and one female, with a median age of 59 years, with no ICI therapy. Reported causes of death in the control group included cardiogenic shock, thromboembolic or hemorrhagic events, aortic dissection, and multiple‐organ failure. From the available cohort, a subset of cases with sufficient tissue availability, appropriate tissue quality, and comparable anatomical region was selected for dual‐immunofluorescence analysis. For quantification, three ICI‐treated cases and three control cases were included. For each case, brain tissue was sectioned and processed for dual immunofluorescence staining using the indicated marker combinations. Three tissue sections per case were stained for each marker combination. From each stained section, we randomly selected three to four non‐overlapping ROI that were imaged using confocal microscopy and further used for 3D algorithm‐based volumetric quantification. Primary antibodies include rabbit anti‐CD16/Fcgr3 (1: 500, Fortis Life Science, Cat# A700‐163‐T, BLR163J), mouse mAb TREM2 (1:500, Cell signaling Technology, Cat# 29715T, E4F5G), rabbit anti‐GFAP (1:400, Biolegend, Cat# 840001, PRB‐571C), mouse alpha‐1‐antichymotrypsin or anti‐SERPINA3 (1:500, Mybiosource.com, Cat# MBS439876, SERPINA3), mouse anti‐human SOX10 (1:500, Mybiosource.com, Cat# MBS439095), mouse anti‐myelin Basic protein (MBP) mAb (1:500, Origene, Cat# SM1453S, Clone ID: 22). Secondary antibodies include goat anti‐rat Alexa Fluor 647 (Abcam, Cat# ab150159; RRID: AB_2566823), goat anti‐rabbit Alexa Fluor 488 (FisherSci, Cat# a11008; RRID: AB_143165), and goat anti‐mouse Alexa Fluor 647 (Abcam, Cat# ab150115; RRID: AB_2687948), Goat anti mouse AF 488 (FisherSci, Cat# A‐11001; RRID: AB_2534069).

For the dual‐immunofluorescence staining, formalin‐fixed, paraffin‐embedded (FFPE) human frontal cortex sections were processed using a standard immunofluorescence protocol. Briefly, Slides were first deparaffinized by incubation in Histo‐Clear for 20 min, followed by sequential rehydration in 100%, 90%, and 70% ethanol for 3 min each, and a final rinse in distilled water for 1 min. Antigen retrieval was performed by immersing slides in antigen retrieval solution (citrate buffer, 10 mm, pH 6) within a glass chamber and heating in a microwave for 4 min (optimized), ensuring boiling for at least 3 min. This heating–cooling cycle was repeated once, after which slides were allowed to cool completely. Slides were then gently wiped to remove excess retrieval solution, washed under running distilled water for 10 min, and equilibrated in 1 × TBST for two washes of 5 min each. For blocking, sections were incubated in a humidified chamber with blocking buffer containing 10% goat serum and 5% BSA prepared in 1 × TBST for 1 h and 30 min at room temperature. Primary antibodies were diluted as above in incubation buffer (1% BSA, 1% goat serum in 1 × TBST) and incubated overnight at 4°C. Slides were subsequently washed three times in 1 × TBST for 5 min each and incubated with the appropriate secondary antibodies diluted 1:500 in the same incubation buffer for 60 min at room temperature. Following secondary incubation, slides were rinsed in distilled water for 1 min and washed three times in 1 × TBST for 10 min each. Nuclear staining was performed using DAPI made in PBS for 10 min (1 µmol/L, FisherSci, Cat# D1306, RRID: AB_2629482), followed by a final wash in 1 × TBST for 10 min. Sections were mounted using a mounting medium (Vectashield Cat# H‐1000‐10) and stored protected from light until imaging.

### 2.6 Conditional T Cell Depletion and Combi‐ICI Treatment

T‐DTR mice (Cd4‐Cre^±^ DTR^+/−^) were generated by crossing of CD4‐Cre (JAX# 017336: Tg(Cd4‐cre)1Cwi/Bflu) mice with B6‐iDTR (JAX#007900: C57BL/6‐Gt(ROSA)26Sortm1(HBEGF)Awai/J). Because of the expression of *Cd4* during the double‐positive (CD4^+^, CD8^+^) stage of thymic selection, T‐DTR mice express diphtheria toxin receptor (DTR) selectively in both CD4^+^ and CD8^+^ T lymphocytes. To achieve robust T cell depletion, T‐DTR mice were injected with diphtheria toxin DT (100 ng, once daily for 2 days) 2 days before or 7 days after Combi‐ICI treatment to determine the impact of T cell ablation on microglial activation state.

We verified the T cell depletion efficiency in T‐DTR mouse spleen using FACS. Briefly, spleen samples were mechanically ground against 70 µm cell strainers (Miltenyi Biotec, catalog #130‐110‐916) over 50 mL conical tubes and washed with RPMI containing 2 mm EDTA. RBC lysis was performed by resuspending cell pellets in pre‐chilled ammonium‐chloride‐potassium (ACK) lysis buffer and incubating on ice for 1 min. ACK was quenched with heat‐inactivated (HI) FBS, then washed with RPMI + 10% HI FBS. Single‐cell suspensions of splenocytes were incubated with Fc receptor‐blocking antibody (anti‐CD16/CD32, clone 93, BioLegend, Cat. #101320) for 15 min at 4°C, followed by surface staining for 30 min at 4°C in blocking buffer (PBS with 10% FBS). Cells were stained with antibodies against CD45 (clone 104, Invitrogen, Cat. #12‐0454‐83), NK1.1 (clone PK136, BioLegend, Cat. #108774), and TCRβ (clone H57‐597, BioLegend, Cat. #109241), each used at a 1: 500 dilution. After staining, cells were washed with FACS buffer (PBS supplemented with 1% FBS) and acquired on a NovoCyte Quanteon flow cytometer (Agilent). Data were analyzed using NovoExpress software (v1.6.3).

WT (C57Bl6) and T‐DTR mice (5–6 months old) were divided into 6 groups: WT + ITC, WT + ICI (anti‐PD‐1, anti‐CTLA‐4 for three weeks as described above), T‐DTR mice receiving DT (100 ng/day for 2 days, IP) either 2‐days prior to or 7 days after the initiation of ICI injections, and T‐DTR mice receiving DT or PBS. 72 h after completion of ICI treatments, mice were humanely euthanized, and PFA‐fixed brains were collected for microglial activation analysis. Dual immunofluorescence staining was conducted to evaluate microglial activation (CD68‐IBA1) as described previously [20, 21].

### 2.7 Confocal Microscopy and 3D Algorithm‐Based Volumetric Quantification

A laser‐scanning confocal microscope (Nikon Eclipse Ti2 AX) was used to image the immuno‐stained brain sections through 24–25 µm mouse brain sections (0.5 µm thickness per z stack) at voxel size 0.428 × 0.428 × 0.500 and in 2048, 2048 and 29 *xyz* resolution and 3 µm for human brain tissues (0.158 µm thickness per z stack) at a voxel size of 0.431 × 0.431 × 0.120 µm and at 1024 × 1024 × 27 *xyz* to obtain 19–20 optical slices and to render high resolution images for the volumetric quantification of immunoreactivity. Acquisition of confocal z‐stacks and 3D algorithm‐based unbiased volumetric quantification of immunofluorescence (refractive Index 1.5, distance 6.60 µm, gamma correction 0.950, and baseline subtraction 150 AU) were carried out as described in detail previously [20, 21]. We created and quantified 3D volume surfaces for each marker of interest. Volumetric analysis and co‐localization of dual‐stained tissues for Fcgr3‐TREM2, Sox10‐MBP, GFAP‐Serpin3n, and CD68‐IBA1 were determined and batch‐analyzed using Imaris (Oxford Instruments) as described previously [20, 21]. All IHC images were uniformly processed using the same parameters to ensure unbiased analysis. For human brain tissues, we included three postmortem cortical samples per group from both ICI‐treated patients and non‐cancer controls. Three sections from each tissue sample were stained for immunofluorescence analysis. We analyzed 3–4 ROIs per section for volumetric quantification of immunoreactivity using markers for microglia, astrocytes, and oligodendrocytes.

### 2.8 Statistical Analysis

All data were expressed as the mean ± SEM. Statistical analyses of immunohistochemical data were conducted using two‐way ANOVA, *t*‐test (non‐parametric), and nested *t*‐test (GraphPad Prism, v8.0. Specifically, for human patient brain tissues, super‐plots were generated using data from three patients per group (non‐cancer controls and ICI‐treated patients), and a nested *t*‐test was performed to determine statistical significance. For gene expression analysis, the log2 fold change (magnitude of expression difference between conditions) and the −log10 of the adjusted *p*‐value (statistical significance after multiple testing correction, Benjamini–Hochberg FDR) were used.

## 3 Results

### 3.1 Hippocampal RNA Sequencing Reveals Modest ICI Therapy‐Driven Changes in Melanoma Burden‐Associated Neuroinflammatory Pathways

We designed the current study based on our previous findings of Combi‐ICI‐related hippocampal‐dependent cognitive impairments and neuro‐immune dysregulation [16], using a clinically relevant melanoma cancer‐survivor model. This syngeneic model (murine D4M‐UV2 cells) treated with a combination of CTLA‐4 and PD‐1 blockade provided the rationale for including four groups in the present transcriptomic study: Control, Control + ICI, Melanoma, and Melanoma + ICI. This design also allowed us to distinguish neuroimmune changes associated with ICI treatment alone, melanoma burden alone, and the combined impact of melanoma and ICI treatment. First, to define molecular networks associated with ICI‐related neuroinflammation and alterations in the hippocampus at the whole‐tissue level, we performed bulk RNA sequencing on hippocampal tissue collected 3 days after completion of ITC or Combi‐ICI (αCTLA‐4 + αPD‐1) treatments (Figure 1A). Differential expression analysis was conducted across three pairwise contrasts: Control versus Melanoma, Melanoma versus Melanoma + ICI, and Control + ICI versus Melanoma + ICI (Figure 1B). For all the data presented in the results, “Combi‐ICI” is referred to as “ICI” for simplicity. Melanoma‐bearing mice exhibited a distinct transcriptional signature relative to controls, consistent with the paradigm that peripheral tumor burden is associated with altered homeostasis of neuroimmune networks [22]. In contrast, ICI‐treated melanoma‐bearing mice showed widespread downregulation of genes compared with the Melanoma group, suggesting that ICI may partially attenuate tumor‐burden associated immune alterations in the brain. Notably, the Melanoma + ICI group still displayed a substantial number of upregulated genes compared with the Control + ICI group, indicating that tumor‐associated transcriptional activation persists in the hippocampus despite ICI therapy. A complementary three‐way Venn diagram further revealed unique patterns (Figure 1C). The Control versus Melanoma comparison contained seven uniquely upregulated genes (*Adora2a, Fosb, Htr1b, Lcn2, Sult1a1, Fmo2, and Acer2*), with an additional three genes shared with the Control + ICI versus Melanoma + ICI group (*Adora2a, Cd4, and Lcn2*). Conversely, the Melanoma + ICI comparison contained four uniquely upregulated genes (*Htr7, Nog, Glra3, and Gm12371)*. Furthermore, we compared the selected gene transcripts across all experimental groups. Notably, *Ctsd* (a microglial lysosomal enzyme involved in antigen‐processing pathways) was highly expressed in both the Melanoma and Melanoma + ICI groups (Figure 1D). In addition, the MHC‐II‐associated microglial genes *H2‐eb1, H2‐ab1*, and *H2‐aa* were moderately expressed but showed pronounced upregulation in the Melanoma + ICI group, suggesting a shift of microglia toward an immune‐activated state. Similarly, *Darpp‐32* (a neural signaling and plasticity‐related gene) was strongly upregulated in the Melanoma + ICI group, indicating that ICI therapy may modulate gene expression associated with neural plasticity.

**FIGURE 1:**
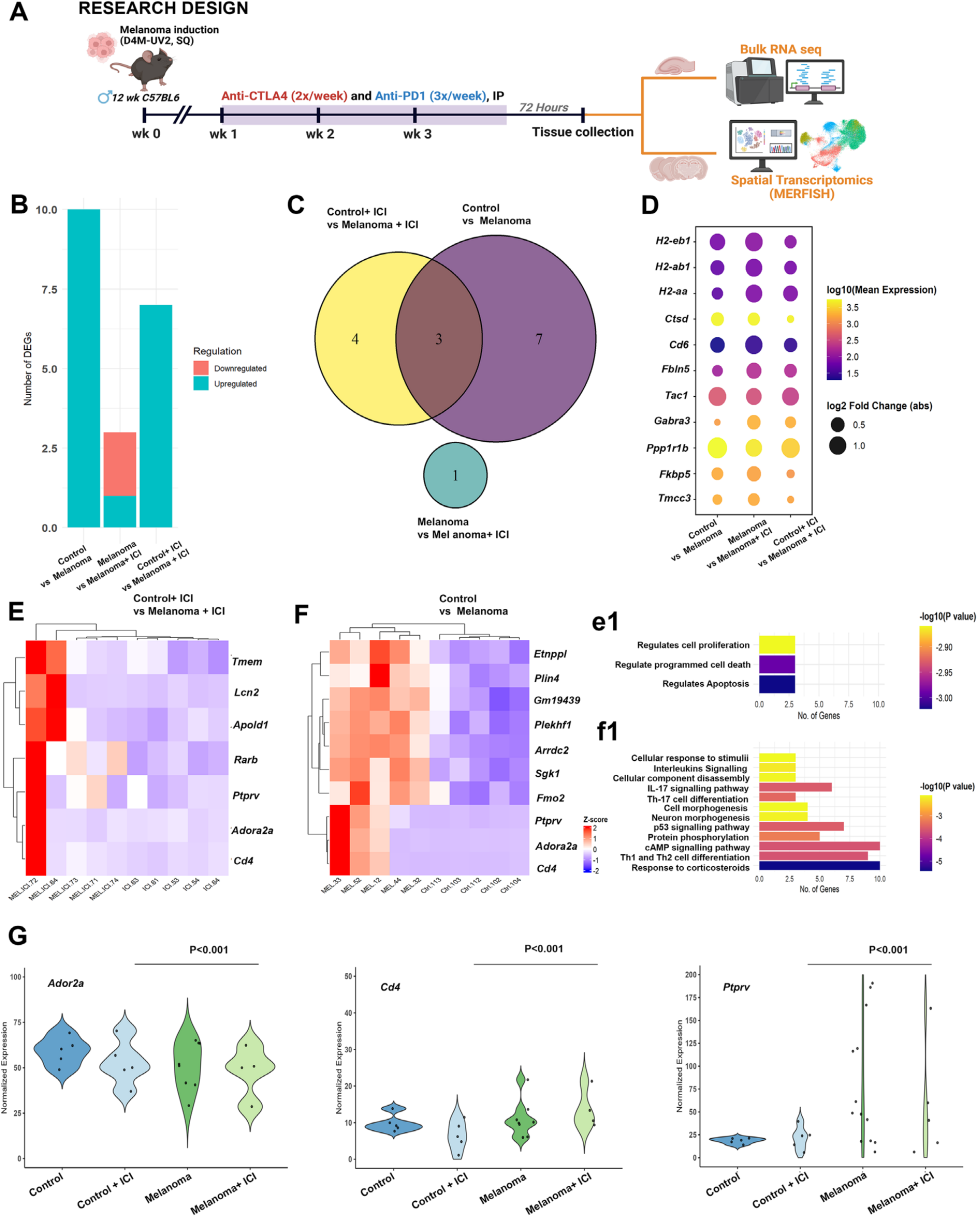
Bulk RNA sequencing of hippocampus post‐ICI treatment. (A) Experimental design: Two‐month‐old C57Bl/6 WT male mice received either sham treatment or melanoma induction (D4M‐UV2 cells; 1 × 10^6^ cells/animal, subcutaneous bilateral injections). Seven days later, mice received either combination immune checkpoint inhibitors (Combi‐ICI) anti‐CTLA‐4 (1 mg per dose, three times weekly, IP) and anti‐PD‐1 (200 µg per dose, twice weekly, IP) or vehicle for three weeks. On day 28, hippocampal tissue was microdissected for bulk RNA‐seq and MERFISH, respectively. (B)Stacked bar plot showing the number of differentially expressed genes (DEGs) in each group. (C) Venn diagram with corresponding functional enrichment analysis of genes upregulated across the groups. Statistically significant analytes among Control, Melanoma, Control ICI, and Melanoma ICI groups are indicated (^#^ *P *< 0.1). (D) Bubble plot depicting dysregulated cell‐type markers in the melanoma ICI group. Bubble size reflects fold change, while color indicates gene expression levels. (E,F) Heatmaps and corresponding enriched pathways showing fold changes and expression patterns of upregulated genes across Control, Melanoma, and Melanoma ICI groups. (e1–f1) Functional enrichment analysis of upregulated genes from Control versus Melanoma and Control ICI versus Melanoma ICI was performed using Metascape based on their predicted target genes. Here, the *x‐*axis represents the number of genes from the dataset associated with each enriched pathway, and the *y‐*axis lists the corresponding biological processes or pathways identified through enrichment analysis.  The color scale indicates the statistical significance of enrichment expressed as −log_10_(*p‐*value). (G) Violin plot showing commonly upregulated genes in both the Control + ICI versus Melanoma + ICI and Control versus Melanoma comparisons. These genes showed strong upregulation and high expression levels across both conditions. Data are derived from *N* = 4 mice per group.

Next, we examined transcriptional changes associated with melanoma and ICI treatment. In the Melanoma + ICI group, the heatmap revealed strong upregulation of several genes (Figure 1E), including *Tmem252, Lcn2, Apold1, Rarb*, Ptprv*, Adora2a, and Cd4*, compared to the Control + ICI group. Pathway enrichment analysis indicated activation of processes such as negative regulation of cell population proliferation and programmed cell death (apoptosis), suggesting that ICI treatment further enhances neuroimmune activation in the presence of melanoma (Figure 1e1). Similarly, in the Melanoma group, a higher fold‐change and statistically significant upregulation was observed in genes including Ptprv*, Adora2a, Cd4, Etnppl, Plin4, Fmo2, Sgk1, Plekhf1, and Arrdc2* compared to the Control group (Figure 1F). DEG‐associated pathways in this comparison included T helper cell activation, interleukin (IL)‐17 signaling, and broader interleukin‐mediated immune pathways, reflecting immune activation and T cell‐associated transcriptional responses in melanoma‐bearing mice (Figure 1f1). In contrast, direct comparison of Melanoma with Melanoma + ICI did not identify any significantly differentially upregulated genes under set statistical criteria (Figure S2).

As shown in the violin plot (Figure 1G), three genes (*Adora2a, Cd4, and Ptprv*) were significantly upregulated (*p* < 0.001) in both the Control + ICI versus Melanoma + ICI and Control versus Melanoma comparisons (Figure 1F). These genes exhibited strong upregulation and high expression levels across both conditions. *Adora2a* (adenosine A2A receptor) is known to mediate microglial activation [23], while *Cd4* is a canonical marker of helper T cells. PTPRV, a tyrosine phosphatase, acts as a potent inhibitor of cell proliferation when highly expressed [24]. Together, these findings indicate that the commonly upregulated genes form a core melanoma‐driven transcriptional program that persists post‐ICI treatment. Importantly, neuroinflammation triggered by melanoma burden was not reversed by ICI therapy and was sustained in the hippocampal microenvironment post‐ICI treatment.

### 3.2 Spatial Transcriptomics Reveals Region‐Specific Cellular Organization and Transcriptional Diversity Across Hippocampal Subfields

To identify cell‐type‐specific transcriptional alterations and regional patterns of ICI‐related neuroinflammation, we designed a custom 300‐gene MERFISH panel (Table S1) representing surrogate markers of inflammation, microglia, astrocytes, oligodendrocytes, neurons, synapses, and T and B cells. The targeted spatial transcriptomic profiling was performed on hippocampal sections from four experimental groups of adult WT (C57Bl6/J) male mice: Control, Control + ICI, Melanoma, and Melanoma + ICI (as in Figure 1A). To characterize spatial organization, we first generated Leiden cluster‐based spatial maps using the cell‐by‐gene expression matrices of brain regions of 2–3 mice per group. High‐quality cell data from hippocampal regions were obtained across all experimental groups: 10 500 from the Control, 14 100 from the Control + ICI, 10 000 from the Melanoma, and 12 500 from the Melanoma + ICI groups, across two to three independent biological replicates per group (Table S2). Cells were normalized, log‐transformed, clustered using graph‐based Leiden clustering, and visualized on their original spatial coordinates to identify spatially organized transcriptional domains across the tissue. Spatial maps demonstrated that microglial, astrocyte‐enriched, oligodendrocyte/myelin‐enriched, and T cell‐enriched populations were distributed across annotated hippocampal subregions at single‐cell segmentation resolution. Heatmaps and corresponding enriched pathways showing fold changes and expression patterns of upregulated genes across Melanoma and Melanoma + ICI groups are shown in Figure S2.

To complement spatial maps, we generated a heatmap to assess the relative percentage of predicted cell types across experimental groups, providing an overview of group‐specific shifts in cellular composition (Figure S3). As expected, excitatory neurons represented the largest cell population across all groups. Specifically, the Melanoma group showed the highest proportion of excitatory neurons across the datasets, whereas the Melanoma + ICI group showed a reduction, suggesting treatment‐associated changes in neuronal cell representation. In parallel, oligodendrocytes were prominently represented in the Control and Melanoma groups. In contrast, the ICI‐treated groups, Control + ICI and Melanoma + ICI, showed a marked reduction in oligodendrocyte proportions compared with their respective non‐ICI groups. Astrocytic and microglial genes were expressed at levels below 10% across all datasets, while T and B cells represented only a very small fraction (0.3%–0.9%) of the identified cell population. Additionally, a high proportion of unclassified, low‐confidence cells were observed across all datasets. This suggests that a large fraction (40%–50%) of cells did not strongly express the selected genes or may have had low transcript counts. Furthermore, MERFISH‐based spatial transcriptomic analysis of the hippocampus resolved distinct neuronal and non‐neuronal cell populations across major subregions, including CA1, CA3, dentate gyrus, and subiculum (Figure 2A). Furthermore, unsupervised clustering revealed the major neuronal and glial cell populations, including excitatory neurons, inhibitory neurons, and non‐neuronal cells, establishing a spatially resolved atlas for evaluating treatment‐associated changes.

**FIGURE 2:**
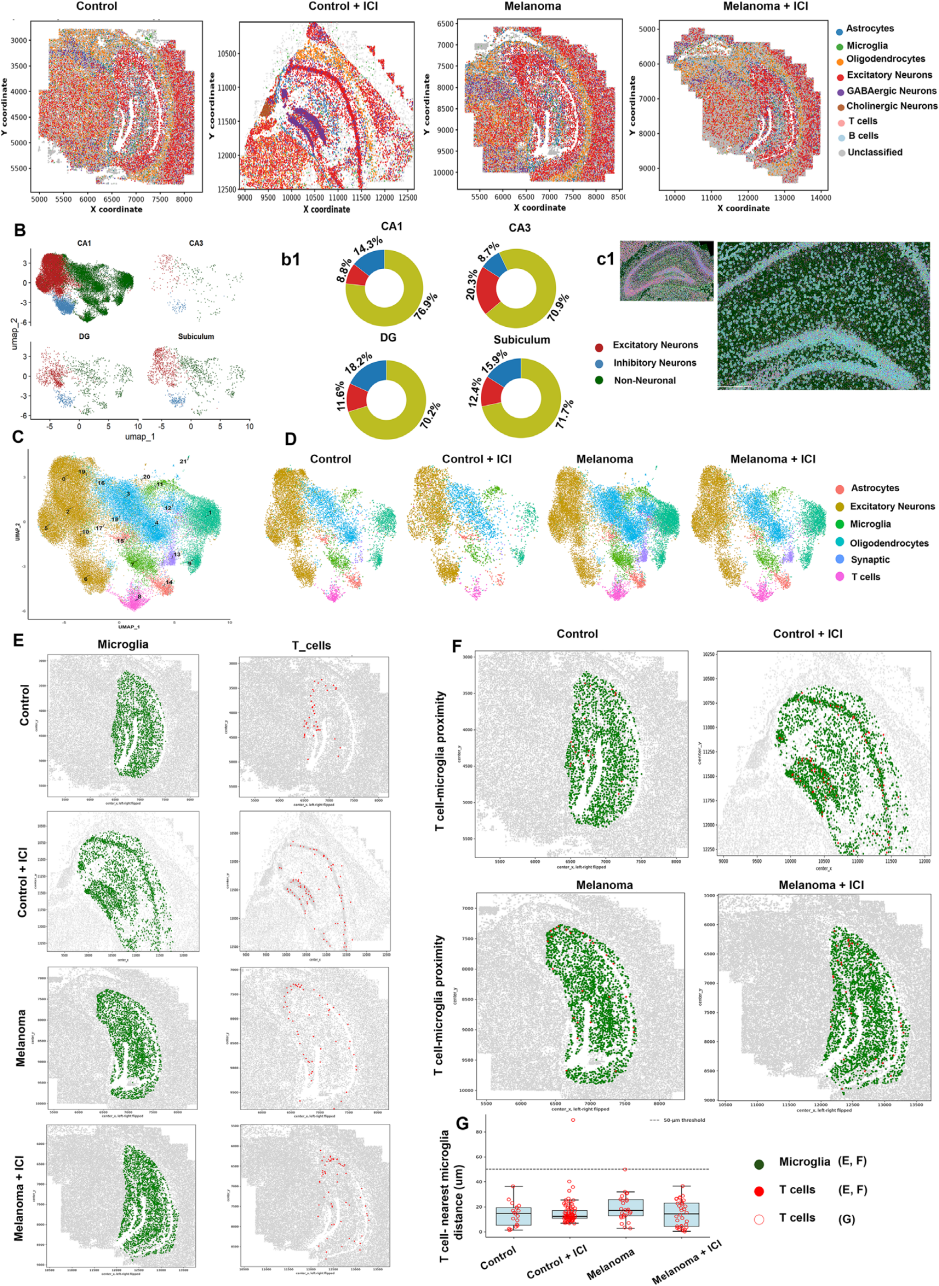
Spatial transcriptomic analysis of hippocampus post‐ICI treatment. (A) Spatial distribution and relative abundance of predicted hippocampal cell populations across treatment groups. MERFISH cell‐centroid coordinates were used to spatially map predicted cell types in Control, Control + ICI, Melanoma, and Melanoma + ICI groups. Cell identities were assigned using log‐transformed marker‐expression scores for astrocytes, microglia, B cells, T cells, oligodendrocytes, excitatory neurons, GABAergic neurons, and cholinergic neurons. Cells with no detectable marker score were classified as low confidence. (B) Uniform Manifold Approximation and Projection (UMAP) embeddings for each region (CA1, CA3, dentate gyrus, and subiculum). (a1) Donut plots showing the proportional distribution of excitatory, inhibitory, and non‐neuronal cell populations across hippocampal subregions (CA1, CA3, dentate gyrus, and subiculum). (C) UMAP visualization shows 21 clusters representing over 7 annotated cell types, following integration across all the experimental groups. (c1) Visualization of nuclei, segmented cell boundaries, and spatially mapped transcripts via MERSCOPE software. (D) UMAP projections stratified by the experimental group dataset revealed clear differences among Control, Control + ICI, Melanoma, and Melanoma + ICI groups. (E) Spatial proximity mapping of T cells and microglia across the dataset. Cell types were assigned using marker‐based expression scores, with T cells and microglia defined by markers as per the gene list. (F) Spatial mapping and nearest‐neighbor proximity analysis of T cells and microglia across treatment groups. Nearest‐neighbor analysis was used to calculate pairwise spatial distances between cell populations. (G) Box plot showing the median Euclidean nearest‐neighbor distance (50 µm) for T cell–microglia spatial relationships within the hippocampal region. Boxes indicate the interquartile range (25^th^–75^th^ percentile), with the central black line representing the median. Individual red dots represent the nearest neighbor distance from each hippocampal T cell to its nearest microglial cell. The nearest neighbor distance was calculated based on a 50 µm proximity threshold. Data are derived from *N* = 3 mice per group.

To assign biological identities to the transcriptionally defined clusters, we combined cluster‐specific marker genes with a curated gene‐class annotation table (Table S4). To visualize the distribution of excitatory, inhibitory, and non‐neuronal cell populations across hippocampal subregions, we generated Uniform Manifold Approximation and Projection (UMAP) embeddings for each hippocampal subregion (CA1, CA3, dentate gyrus, and subiculum, Figure 2B). The regional IDs corresponding to these subfields were obtained from the Allen Brain Atlas (e.g., CA1 = 382, CA3 = 400, DG = 402, Subiculum = 418). Each point in the UMAP represents a single cell (or voxel‐averaged expression profile), colored by its annotated cell type, including excitatory, inhibitory, or non‐neuronal. The resulting plots highlight region‐specific segregation and transcriptional diversity within the hippocampus. To visualize the proportional distribution of excitatory, inhibitory, and non‐neuronal cell populations across hippocampal subregions (CA1, CA3, dentate gyrus, and subiculum), we created donut plots (Figure 2b, 1). Together, non‐neuronal cells comprise the largest population in the CA1 region of the hippocampus, followed by all excitatory neurons and then the inhibitory neurons. Furthermore, using ROI‐based annotations, we assigned MERFISH‐profiled cells to CA1, CA3, DG, and Subiculum and quantified the distribution of non‐neuronal cell classes across these hippocampal subregions. Cell identities were derived from cluster‐level marker matching, after which regional cell counts were visualized using stacked horizontal bar plots. Among the non‐neuronal populations captured by ROI‐based MERFISH annotation, CA1 contained the highest overall number of cells, narrowly followed by DG, with lower counts in Subiculum and CA3 (Figure S1B). Across all four hippocampal subregions, oligodendrocytes and microglia were consistently detected at appreciable levels, with astrocytes showing relative enrichment in DG. In contrast, T‐cell and T‐cell/Complement‐enriched populations were present at lower frequencies across regions. Overall, these data support region‐specific variation in non‐neuronal cellular composition across the ROI‐defined hippocampal subregions.

To further evaluate our detection accuracy, we first performed an integrated analysis of the MERFISH data. The UMAP visualization shows 21 clusters representing more than 7 annotated cell types after integration across all experimental groups (Figure 2C(c1)). UMAP projections stratified by the experimental group dataset revealed clear changes among the Control, Control + ICI, Melanoma, and Melanoma + ICI groups (Figure 2C). In particular, oligodendrocytes, astrocytes, T cells, microglia, and synaptic function‐associated gene sets showed marked variation across conditions. For example, oligodendrocyte proportions were dramatically reduced in the Control + ICI group (6.38%) compared to the Melanoma and Melanoma + ICI groups (19.2% and 17.4%, respectively). Conversely, synaptic function‐associated gene markers were substantially enriched in the Melanoma group (26.18%) relative to the Control and ICI‐treated groups (Con + ICI and Melanoma + ICI). Similarly, T cells were more abundant in Melanoma (3.14%) and Melanoma + ICI (2.46%) than in animals without cancer (Table S4).

To quantify the spatial organization and proximity between T cells and microglia within hippocampal sections and their subfields, we generated spatial cell‐type maps (Figure 2E–G). This approach allowed us to assess group‐specific differences in T cell–microglia spatial proximity within the hippocampal microenvironment. Using MERFISH‐derived cell‐centroid coordinates, we performed nearest‐neighbor distance analysis with a 50 µm proximity threshold. For hippocampus‐specific analyses, cells located within the combined CA1, CA3, dentate gyrus, and subiculum regions were identified using the corresponding region‐of‐interest coordinates to determine T cell‐microglia interactions. For each source cell, the nearest target cell was identified from the complete target‐cell population within the hippocampus. The directional relationships included T cells as source cells with microglia, astrocytes, or oligodendrocytes as target cells, and microglia as source cells with astrocytes or oligodendrocytes as target cells. Euclidean nearest‐neighbor distances were calculated using the spatial coordinates of the source and target cells as described [25], using the formula:

$$d=\sqrt{{\left(Xsource\;-\;Xtarget\right)}^{2}+{\left(Ysource\;-\;Ytarget\right)}^{2}}\;$$

T cells in the Control + ICI groups showed the shortest median nearest‐neighbor distances to microglia (12.54 µm), followed by Melanoma + ICI (14.40 µm), compared with the Control and Control + ICI groups (Figure 2G). These data suggest that ICI treatment potentially promotes closer spatial association between T cells and microglia, forming local neuroimmune proximity zones. However, hippocampal subfields showed little to no T cell and Microglia interaction across the datasets (Figure S4A).

Neighborhood proximity analysis of T cell–microglia pairs with astrocytes and oligodendrocytes showed a shorter median distance between T cells and astrocytes in the Melanoma + ICI group (17.6 µm, Figure S5A,B). Similarly, the ICI‐treated groups showed shorter microglia–oligodendrocyte and microglia–astrocyte distances Figure 5A,C). The median nearest‐neighbor distances were 18.6 µm in the Melanoma + ICI group and 21.7 µm in the Control + ICI group, respectively, compared with the Control and Melanoma groups (Figure S5A). Together, these findings suggest that ICI therapy differentially alters T cell interactions with the brain's glial population and microglial interactions with other glial neighborhoods within the hippocampus.

### 3.3 ICI Therapy Drives a Coordinated T Cell–Microglial Inflammatory Axis Marked by Microglial Activation and T Cell Responses

To investigate dysregulation of the T cell–microglial axis following ICI treatment as a potential driver of ICI‐related irAEs, we used a clustering approach to identify spatial gene expression across hippocampus sections (Figures 3, 4). Cell‐specific canonical gene markers were identified and re‐clustered with Seurat's graph‐based clustering, yielding multiple transcriptionally distinct clusters that were subsequently relabeled as M1–M16 for Microglia and T1‐T16 for T cells (Figures S6,S7). To assess the relative distributions of these subpopulations under different experimental conditions, UMAP plots were split by experimental group (Control, Control + ICI, Melanoma, and Melanoma + ICI; Figures 3A and 4A). Each dot represents a single microglial cell, and cells are colored according to their assigned cluster identity (e.g., M1–M16, T1‐ T16). This coloring highlights transcriptionally distinct microglial subpopulations and enables direct visual comparison of how the relative abundance and distribution of these clusters vary across experimental conditions. Clustering of the microglial subset using the integrated microglial/T cell Seurat object  at resolution 1.0 generated 16 microglial/T cell clusters, reflecting substantial transcriptional diversity. We selected this resolution because it captured meaningful microglial subpopulations while avoiding over‐fragmentation and maintaining cluster stability. Heatmap analysis of microglial/T cell marker genes further supported the biological relevance of these clusters, revealing cluster‐specific gene expression profiles across activation‐associated microglial/T cell transcripts (Figures S2B,S3B).

**FIGURE 3:**
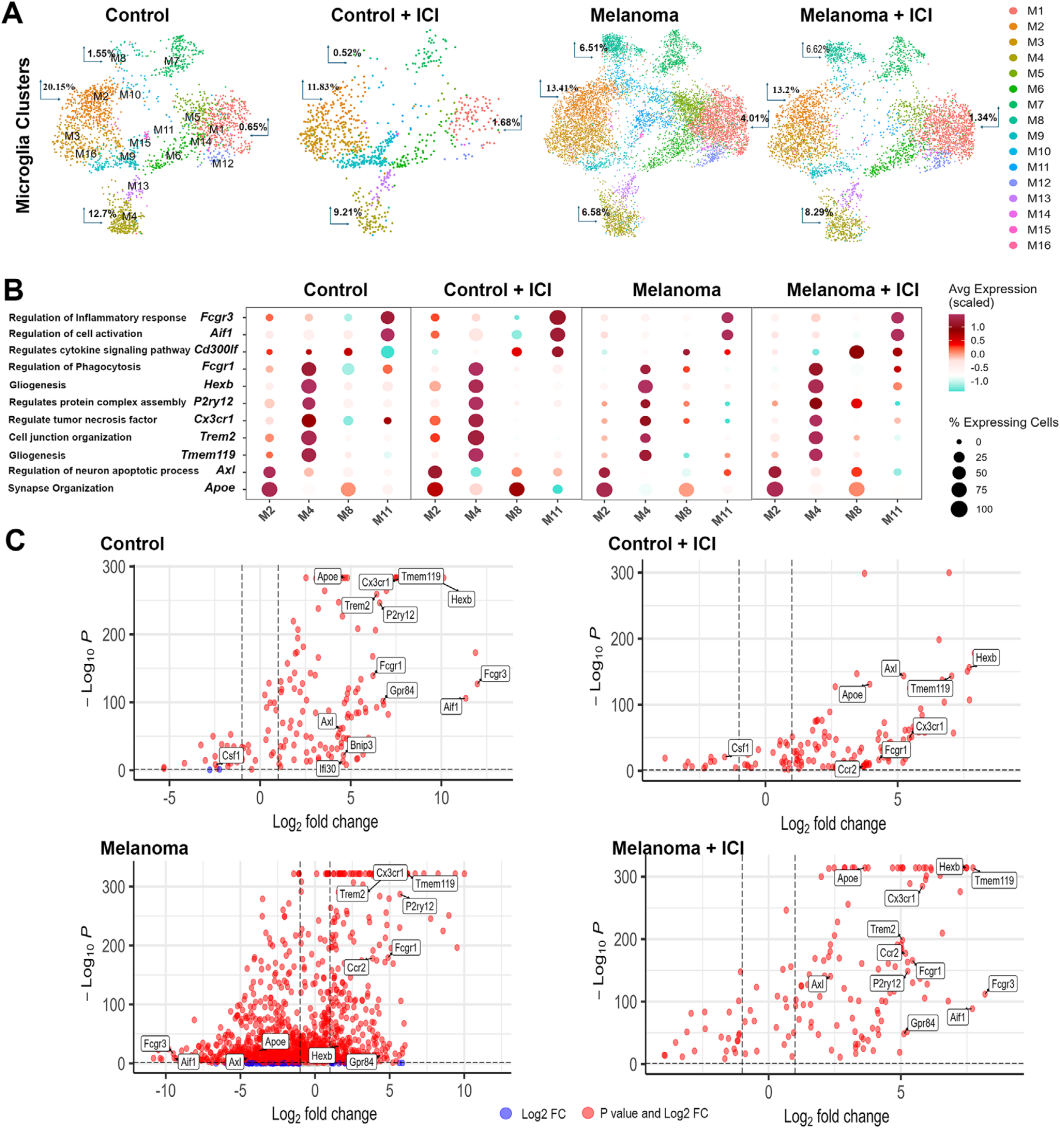
Gene expression analysis of the microglial cluster. (A) Split UMAPs display gene clusters across Control, Control + ICI, Melanoma, and Melanoma + ICI group datasets. (B) Bubble plots depicting the distribution and expression of genes within clusters. Bubble size represents the percentage of cells expressing the specific gene. The bubble color indicates the average expression level of that gene within the corresponding cluster. (C) Volcano plots depicting differential gene expression across the experimental group, with each point representing a gene. The *x*‐axis corresponds to the log2 fold change (magnitude of expression difference between conditions), while the *y*‐axis corresponds to the −log10 of the adjusted *p*‐value (statistical significance after multiple testing correction, Benjamini–Hochberg FDR). Downregulated genes are further to the left, while upregulated genes are further to the right. Higher −log_10_ *P*‐values indicate higher statistical significance in gene expression. Significance thresholds used were: adjusted *p* < 0.05 and log2 fold change > 1. Red: Strongly upregulated, high significance. Blue: Strongly downregulated, high significance. Data are derived from *N* = 3 mice per group.

**FIGURE 4:**
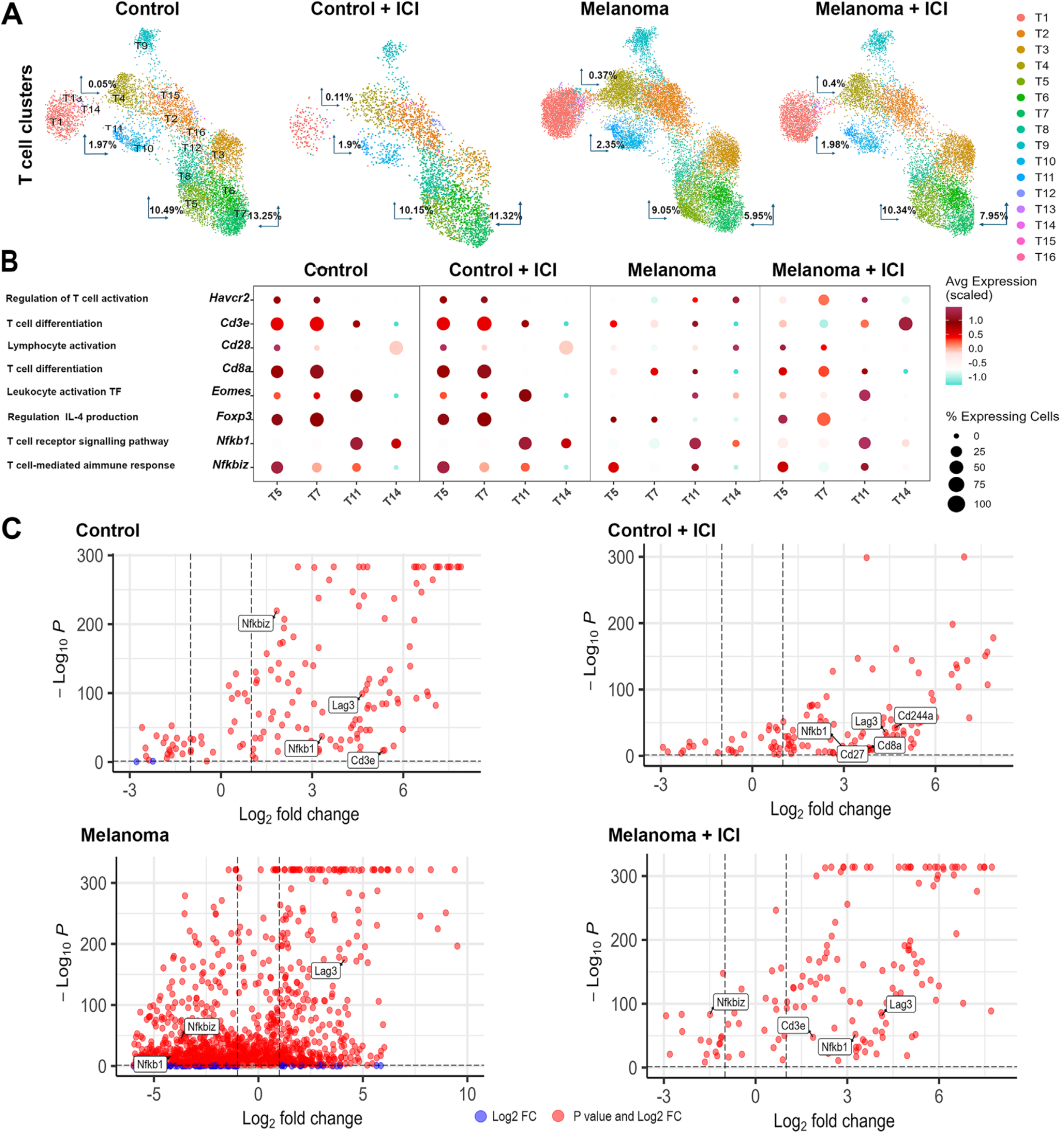
Gene expression analysis of the T cell cluster. (A) Split UMAPs display gene clusters across Control, Control + ICI, Melanoma, and Melanoma + ICI group datasets. (B) Bubble plots depicting the distribution and expression of genes within clusters. Bubble size represents the percentage of cells expressing the specific gene. The bubble color indicates the average expression level of that gene within the corresponding cluster. (C) Volcano plots depicting differential gene expression across the experimental group, with each point representing a gene. The *x*‐axis corresponds to the log2 fold change (magnitude of expression difference between conditions), while the *y*‐axis corresponds to the −log10 of the adjusted *p*‐value (statistical significance after multiple testing correction, Benjamini–Hochberg FDR). Downregulated genes are further to the left, while upregulated genes are further to the right. Higher −log_10_ *P*‐values indicate higher statistical significance in gene expression. Significance thresholds used were: adjusted *p* < 0.05 and log2 fold change > 1. Red: Strongly upregulated, high significance. Blue: Strongly downregulated, high significance. Data are derived from *N* = 3 mice per group.

### 3.4 Microglial Activation, a Key Driver of Pro‐Inflammatory Dysregulation, Following ICI Therapy

To further investigate melanoma and ICI‐related microglial (dys)regulation, we analyzed individual microglial cell clusters to link the spatially resolved gene function. Pairwise Fisher's exact tests demonstrated that microglial clusters M2, M4, M8, and M11 exhibited significant alterations across experimental conditions (Table S5). Based on unbiased statistical criteria, we prioritized M2, M4, M8, and M11 because they showed the most consistent and biologically interpretable shifts across tumor‐ and ICI‐associated conditions. Clusters M2, M4, M8, and M11 were markedly enriched in Melanoma and Melanoma + ICI compared with Control, with the strongest effects observed for clusters M4, M8, and M11. These clusters showed highly significant differences across nearly all melanoma‐related comparisons. In contrast, Control versus Control + ICI comparisons revealed more modest but still significant shifts for clusters M2, M4, M8, and M11 (with a slight change in M7), indicating that ICI alone can alter microglial composition independent of tumor burden.

To quantify microglial cluster composition (M1–M16) across groups, we calculated the per‐sample proportion of cells assigned to each cluster and summarized these distributions using 100% stacked bar plots (Table S5, Figure S6A). Further characterization of the functional identities of each cluster highlighted their defining marker genes and expression patterns across datasets (Figure 3B). Briefly, in the Control + ICI group, clusters M4 and M11 contained a higher proportion of cells, with ∼100% of cells expressing *Aif1* (a microglial activation marker) [26] and *Fcgr3* (a regulator of microglial polarization) [27]. The concurrent upregulation of *Aif1* and *Fcgr3* suggests that these clusters shift toward an activated, pro‐inflammatory state, consistent with Fcgr3's known role in modulating inflammatory responses under ICI treatment. Approximately 75% of genes expressed in cluster M4 across the Control, Control + ICI, and Melanoma + ICI groups corresponded to canonical homeostatic microglial markers, including *Trem2, Tmem119, P2ry12, Cx3cr1, and Hexb*. The relative loss of this homeostatic signature in the Melanoma group indicates a disruption of baseline microglial maintenance functions. Similarly, cluster M8, which expanded in both vehicle‐ and ICI‐treated melanoma groups compared with Control, contained a high proportion (∼75%) of cells expressing *Apoe*. In contrast, the larger M8 cluster in the Melanoma + ICI group was enriched for *Cd300lf*, suggesting lower neuroinflammatory activity [28] and a possible role for microglia in promoting neurological recovery following ICI therapy.

To investigate gene‐level changes complementary to the cluster‐based observations, we next sought to identify individual genes that were significantly altered across datasets. We performed differential expression analysis of microglial populations and visualized the results using volcano plots. As shown in the figure (Figure 3C), the key homeostatic microglial marker *Hexb* was strongly upregulated in the Control, Control + ICI, and Melanoma + ICI groups, but weakly upregulated in the Melanoma group. This pattern suggests that in the Melanoma group, microglial homeostatic programs are disrupted, whereas ICI partially restores or preserves some of these features. Similarly, *Gpr84*, expressed by activated microglia, was upregulated under pathological conditions, consistent with previous reports [29]. In line with these findings, our results demonstrate that *Gpr84* was significantly upregulated in both the Melanoma and Melanoma + ICI groups, supporting its role as an inflammatory amplifier of microglial pathophysiology. Overall, these findings show that Melanoma and ICI treatment dynamically alter microglial states, with evidence of both pro‐inflammatory activation and disruption of homeostatic programs, validating the role of microglia as key mediators of neuroimmune alterations associated with ICI.

### 3.5 ICI Therapy Stimulates T Cell and NF‐κB‐Mediated Inflammatory Responses

To assess the relative distributions of the transcriptionally distinct T cell subpopulations, similar to microglia, UMAP plots were split by experimental groups (Control, Control + ICI, Melanoma, and Melanoma + ICI; Figure 4A). Pairwise Fisher's exact tests revealed condition‐specific alterations across several T cell clusters (Table S6). Cluster T7 exhibited highly significant differences across the datasets. Comparisons of Control versus Control + ICI, Melanoma, and Melanoma + ICI all showed strong shifts (*P* < 0.001). A significant alteration was also observed between Melanoma versus Melanoma + ICI (*P *< 0.001), indicating that T7 abundance is influenced by both melanoma and ICI treatment. Additionally, Cluster T5 showed a moderate shift across the datasets, with significant differences between Control versus Melanoma (adjusted *p* < 0.05) and Melanoma versus Melanoma + ICI (adjusted *p* < 0.01). Cluster T14 showed significant differences between Control versus Melanoma and Control versus Melanoma + ICI (*P* < 0.05). Conversely, Cluster T11 showed no significant differences across all datasets, suggesting no major transcriptional changes between treated and untreated groups.

Additionally, we calculated the proportion of cells per sample for each cluster and visualized their distribution using 100% stacked bar plots (Figure S7A and Table S6). Heatmap analysis of T cell marker genes further supported the biological relevance of these clusters by revealing cluster‐specific expression profiles (Figure S7B). Cluster T14 showed nearly 100% of cells expressing Havcr2 in the Control + ICI group compared to the Melanoma and Melanoma + ICI groups, suggesting enhanced T cell activation following ICI therapy (Figure 4B). Similarly, *Cd3e* (Clusters T5, T7), *Cd28* (Cluster T14), and *Cd8a* (Clusters T5, T7) were expressed by approximately 75% of cells in the Control and Control + ICI groups, whereas their expression was markedly reduced in the Melanoma and Melanoma + ICI groups. In contrast, the *Nfkbiz* gene showed substantial variability across all clusters (T5, T7, T11, and T14) and datasets.

To investigate gene‐level changes complementing the cluster‐based findings, we next examined the expression of key T cell–associated genes across all four conditions (Figure 4C). Differential expression analysis revealed robust upregulation of canonical markers associated with T cell signaling and activation, including *Cd3e, Lag3, Nfkb1, and Nfkbiz* in the Control group, indicating a stable baseline activation and homeostatic regulatory state in resting T cells. In contrast, the Melanoma group displayed a markedly restricted transcriptional profile, characterized by strong downregulation of *Nfkb1* (*P* < 0.05), a pattern consistent with tumor‐driven T cell exhaustion and impaired NF‐κB‐mediated inflammatory signaling [30]. Notably, ICI treatment in the Melanoma + ICI group partially restored T cell activation programs. Genes such as *Nfkb1 and Cd3e* were also expressed at higher levels, while *Lag3* remained highly upregulated, indicating persistent but modulated T cell activation post ICI therapy. The modest downregulation of *Nfkbiz* in this group is consistent with altered NF‐κB regulatory feedback associated with inflammatory activation post ICI therapy [31].

### 3.6 ICI Therapy Alters Astrocyte Homeostatic Markers

We previously reported a modest decrease in the astrocytic marker GFAP following Combi‐ICI treatment [16]; however, the underlying transcriptomic dysregulation remained unclear. Similar to the microglia and T cells, to assess relative distributions of these subpopulations under different experimental conditions, UMAP plots were split by the experimental groups (Control, Control + ICI, Melanoma, and Melanoma + ICI, Figure 5A). Clustering of the astrocyte subset using the integrated astrocyte Seurat object at resolution 1.0 generated 18 astrocyte clusters (Figure S8A), reflecting substantial transcriptional diversity. We selected this resolution because it captured meaningful astrocyte subpopulations while avoiding over‐fragmentation and maintaining cluster stability. Heatmap analysis of astrocyte marker genes further supported the biological relevance of these clusters, revealing cluster‐specific expression profiles (Figure S8B). Pairwise Fisher's exact tests revealed condition‐specific shifts in astrocyte subclusters. Cluster A5 showed significant enrichment in melanoma‐related comparisons (Control vs. Melanoma and Control vs. Melanoma + ICI, *p* < 0.001; Control + ICI vs. Melanoma and Control + ICI vs. Melanoma + ICI, *p* < 0.05). Cluster A8 displayed the strongest alterations, with highly significant differences in all melanoma‐involving contrasts (*P *< 0.001) and a modest but significant change in Control versus Control + ICI (*P* < 0.001). Cluster A13 showed more limited changes, significant only between Control + ICI versus Melanoma and Control+ ICI versus Melanoma + ICI (*P *< 0.05). Cluster A15 showed no significant differences (all *P* ≥ 0.05). Overall, A5, A8, and A13 demonstrated condition‐specific alterations, whereas A15 remained stable across datasets.

**FIGURE 5:**
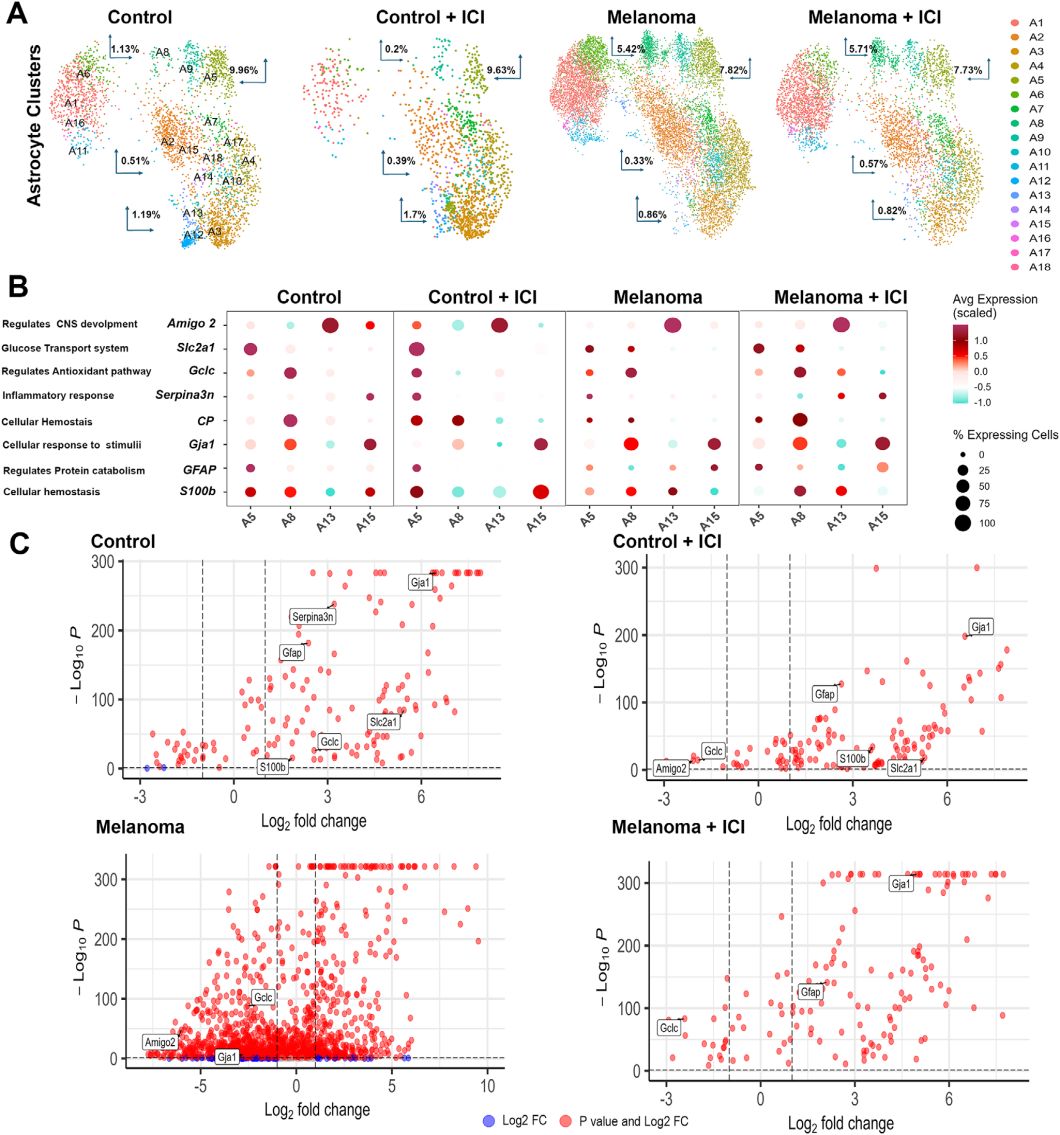
Gene expression analysis of the astrocyte cluster. (A) Split UMAPs display gene clusters across Control, Control +ICI, Melanoma, and Melanoma +ICI group datasets. (B) Bubble plots depicting the distribution and expression of genes within clusters. Bubble size represents the percentage of cells expressing the specific gene. The bubble color indicates the average expression level of that gene within the corresponding cluster. (C) Volcano plots depicting differential gene expression across the experimental group, with each point representing a gene. The *xx*‐axis corresponds to the log2 fold change (magnitude of expression difference between conditions), while the *y*‐axis corresponds to the −log10 of the adjusted *p*‐value (statistical significance after multiple testing correction, Benjamini–Hochberg FDR). Downregulated genes are further to the left, while upregulated genes are further to the right. Higher −log_10_ *P* values indicate higher statistical significance in gene expression. Significance thresholds used were: adjusted *P *< 0.05 and log2 fold change > 1. Red: Strongly upregulated, high significance. Blue: Strongly downregulated, high significance. Data are derived from *N* = 3 mice per group.

To quantify astrocyte cluster composition (A1‐A18) across groups, we calculated the per‐sample proportion of cells assigned to each cluster and summarized these distributions using 100% stacked bar plots (Table S7). Further characterization of the functional identities of each cluster highlighted defining marker genes and their expression patterns across datasets (Figure 5B). In the Control and Control + ICI groups, cluster A5 contained a higher proportion of cells (∼50%) with elevated expression of *Slc2a1* (GLUT1) compared to the Melanoma and Melanoma + ICI groups, although its direct association with melanoma remains to be fully elucidated [22]. Furthermore, the expression and proportion of cells expressing *Gja1* increased across clusters A5, A8, A13, and A15 in the Melanoma and Melanoma + ICI groups relative to Control and Control + ICI, suggesting a role in mediating tumor–astrocyte or tumor–immune interactions (Hu W et al., 2020). Similarly, *GFAP* expression reached ∼75% across all clusters in the Melanoma + ICI group, consistent with astrogliosis following ICI treatment. In cluster A8, ∼75% of cells expressed *Cp* in the Control, Control + ICI, and Melanoma + ICI groups, suggesting restoration of neuroprotective functions after ICI therapy. Interestingly, cluster A15, which did not show significant differences across datasets, contained a high proportion (∼75%) of cells expressing *S100b* in the Control + ICI group, indicating stable expression of this neurotrophic marker across conditions.

We next examined the expression of individual astrocyte markers across datasets (Figure 5C). Differential expression analysis revealed that *Gja1*, a canonical astrocyte marker, was strongly upregulated in the Control (*P *< 0.001, log2FC = 6.49), Control + ICI (*P *< 0.001, log2FC = 6.56), and Melanoma + ICI (*P *< 0.001, log2FC = 4.99) groups, but only moderately altered in the Melanoma group (*P* ≥ 0.05). Similarly, *GFAP* (*P* < 0.001, log2FC = 2.64 in Control + ICI) and *Slc2a1* (*P *< 0.001, log2FC = 5.28 in Control + ICI), markers associated with reactive states and neuroprotective effects [32], were upregulated in Control + ICI and Melanoma + ICI, indicating astrogliosis responses in the context of ICI treatment and tumor presence. Conversely, *Gclc* (*p* < 0.05, log2FC = −2.42 in Melanoma + ICI), a gene linked to antioxidant pathways [33], and *Amigo2* (*p* < 0.001, log2FC = −6.01 in Melanoma + ICI), an anti‐inflammatory marker [34], were downregulated in the Control + ICI and Melanoma + ICI groups, suggesting a dysregulation of astrocyte functional states under ICI conditions. Overall, these results show that ICI therapy modulates astrocyte gene expression, reflecting a balance between homeostatic maintenance and reactive activation.

### 3.7 ICI Therapy Counteracts Melanoma‐Associated Oligodendrocyte Dysregulation

To understand how Combi‐ICI therapy affects myelination and contributes to cognitive impairment, we examined the relative distributions of multiple transcriptionally distinct oligodendrocyte subpopulations, labeled O1–O17, using UMAP plots split by experimental group (Control, Control + ICI, Melanoma, and Melanoma + ICI, Figure 6A). Similar to microglia and astrocytes, oligodendrocyte subsets revealed 17 clusters. Heatmap analysis of oligodendrocyte marker genes further supported the biological relevance of these clusters by revealing cluster‐specific expression profiles (Figure S9A,B). Pairwise Fisher's exact tests revealed condition‐specific alterations in oligodendrocyte subclusters (Table S8). Cluster O1 exhibited the strongest and most widespread shifts in cell count proportions, with highly significant differences in all comparisons involving Control versus Control + ICI, Melanoma, and Melanoma + ICI (all adjusted *p* < 0.001). A significant difference in cell count proportions was also observed in Melanoma (18.75%) and Melanoma + ICI (20.23%) compared with Control (11.5%) (adjusted *p* < 0.05). Cluster O8 showed significant differences between Control versus Melanoma and Control versus Melanoma + ICI (adjusted *P*‐values < 0.001). Cluster O10 displayed significant changes in Control versus Control + ICI and Control + ICI versus Melanoma + ICI (adjusted *P* < 0.05). In contrast, Cluster O16 showed minimal variability, with only a modest difference between Control + ICI and Melanoma + ICI (adjusted *p* < 0.05) and no significant differences between Control and Melanoma. Overall, clusters O1, O8, and O10 demonstrated condition‐specific shifts, whereas O16 remained largely stable across datasets.

**FIGURE 6:**
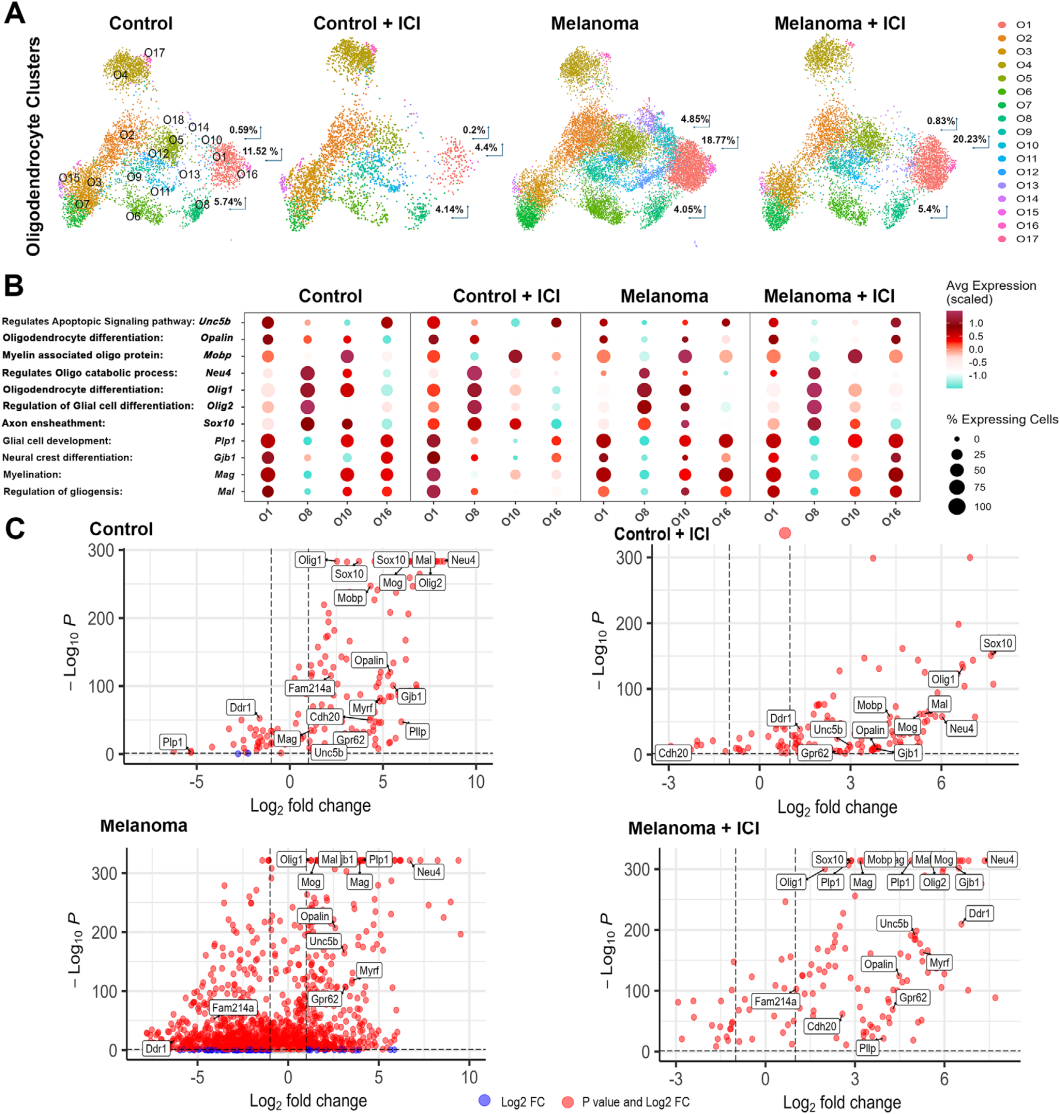
Gene expression analysis of the oligodendrocyte cluster. (A) Split UMAPs display gene clusters across Control, Control +ICI, Melanoma, and Melanoma +ICI group datasets. (B) Bubble plots depicting the distribution and expression of genes within clusters. Bubble size represents the percentage of cells expressing the specific gene. The bubble color indicates the average expression level of that gene within the corresponding cluster. (C) Volcano plots depicting differential gene expression across the experimental group, with each point representing a gene. The *x*‐axis corresponds to the log2 fold change (magnitude of expression difference between conditions), while the *y*‐axis corresponds to the −log10 of the adjusted *p*‐value (statistical significance after multiple testing correction, Benjamini–Hochberg FDR). Downregulated genes are further to the left, while upregulated genes are further to the right. Higher −log_10_ *P*‐values indicate higher statistical significance in gene expression. Significance thresholds used were: adjusted *p* < 0.05 and log2 fold change > 1. Red: Strongly upregulated, high significance. Blue: Strongly downregulated, high significance. Data are derived from *N* = 3 mice per group.

To characterize the composition of oligodendrocyte clusters (O1–O17) across conditions, we calculated the per‐sample proportion of cells assigned to each cluster and visualized these distributions using 100% stacked bar plots (Figure S9A and Table S8). Cluster O1 showed notable variation across datasets, driven in part by differential expression of *Unc5b*. Approximately 75% of cells in the Control and Control + ICI groups expressed Unc5b, compared with substantially lower proportions in the Melanoma and Melanoma + ICI groups (Figure 6B). Clusters O10 and O16 exhibited higher proportions of *Sox10‐*expressing cells in the Control, Control + ICI, and Melanoma + ICI groups relative to Melanoma alone, indicating altered regulation of the oligodendrocyte‐specific transcription factor *Sox1*0 in disease. Conversely, reduced expression of *Gjb1* within Cluster O1 in both Melanoma and Melanoma + ICI groups suggests impaired CNS myelination and homeostasis under tumor and ICI‐associated conditions [35].

Differential gene expression analysis of oligodendrocyte markers revealed consistent upregulation of core myelin‐associated genes, including *Mag, Plp1, Mog, Mal, and Opalin*, in the Control, Control + ICI, and Melanoma + ICI groups (*P *< 0.001, Figure 6C), indicating preservation of mature oligodendrocyte identity across these conditions. In contrast, the Melanoma group showed a more restricted expression profile, with *Ddr1 and Fam214a* significantly downregulated (both *P* < 0.05). The Control + ICI group exhibited downregulation of only the *Cdh20* gene. This pattern indicates that myelin‐related transcriptional programs remain intact under ICI therapy. Notably, several genes (*Sox10, Myrf, and Unc5b*) were also robustly upregulated in Melanoma + ICI, suggesting that ICI may partially reverse or compensate for melanoma‐induced oligodendrocyte dysfunction. Overall, these results demonstrate that oligodendrocyte gene expression is differentially affected by melanoma and by ICI treatment.

### 3.8 Immunofluorescence Analyses of Postmortem Brains From the ICI‐Treated Patients Identify Dysregulated Glial Homeostasis

To verify whether non‐neuronal gene programs identified in our mouse MERFISH dataset translate to human brains, we selected a panel of cell–type–specific markers shortlisted from MERFISH profiling of mouse brains across experimental groups (Control, Control + ICI, Melanoma, and Melanoma + ICI) and evaluated their expression in frontal cortex from postmortem human brain tissue. Because postmortem brain tissue from cancer patients not exposed to ICI was not available, we compared postmortem tissue from patients receiving ICI treatment (anti‐PD‐1 and Combi‐ICI) for different malignancies to a noncancer, non‐treated control cohort. Using dual immunofluorescence staining, we measured the immunoreactivity of glial cell type–specific markers, including microglial activation (CD16/FCGR3 with TREM2), astrogliosis (GFAP with SERPINA3), and oligodendrocytes (SOX10 with MBP) in the frontal cortex of postmortem patients (Figure 7). Quantification of single and co‐localized immunoreactivity was performed using an in silico 3D algorithm‐based volumetric approach as described previously [20, 21]. Patients treated with anti‐PD‐1 therapy (ICI patients) showed a significant increase in dual‐labeling of CD16/FCGR3 (*P < 0.05*) and SOX10/MBP (*P < 0.001*) immunoreactivity volumes compared with non‐cancer control patient brains (*P*. In contrast, MBP immunoreactivity was reduced by approximately 20% following anti‐PD‐1 treatment compared with non‐cancer controls (*p* < 0.05). GFAP/SERPINA3 co‐localization was 1.5 times higher in non‐cancer control patients compared with ant‐PD‐1‐treated patients (*P < 0.05*). Collectively, these data demonstrate a pronounced disruption of microglial, astrocytic, and oligodendrocyte signatures following ICI treatment, supported by parallel evidence from human brains exposed to anti‐PD‐1 therapy.

**FIGURE 7:**
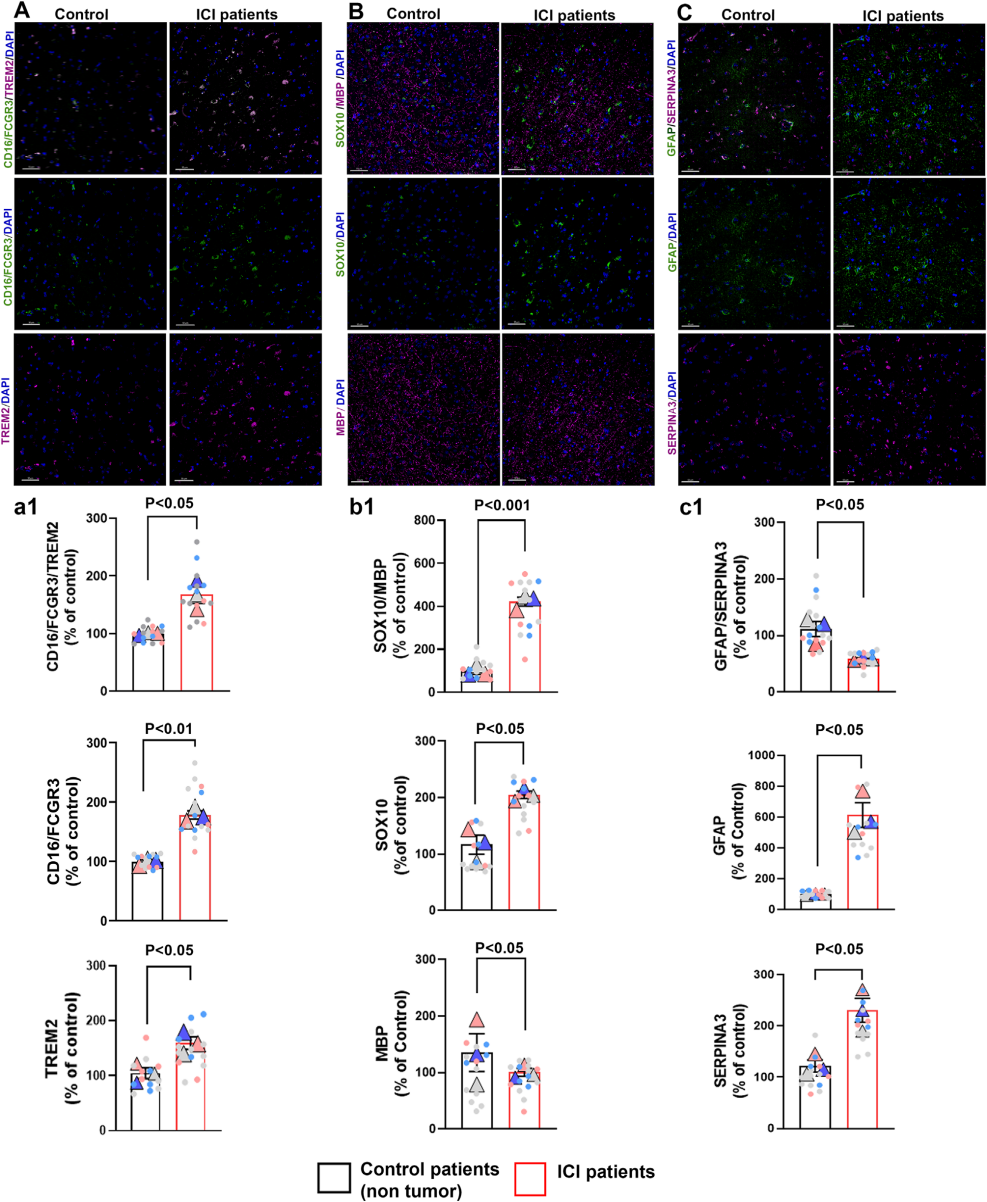
Immunofluorescence analysis of human postmortem brains from ICI‐treated patients. (A–C) Dual immunofluorescence staining for CD16/FCGR3 and TREM2 (microglia), GFAP and SERPINA3 (astrocytes), and SOX10 and MBP (oligodendrocytes and myelin) in frontal cortices from patients receiving ICI treatment for different malignancies compared with a non‐cancer, untreated control cohort (*N* = 3 patients/group). Scale, 50 µm (A–C). (a1–c1) Volumetric quantification of immunoreactivity from 3–4 regions of interest (ROIs) per brain section across three sections per brain, corresponding to the immunofluorescence images shown in (A–C). Quantification shows increased immunoreactivity for microglial‐, astrocyte‐, and oligodendrocyte‐specific gene markers in the cortices of ICI‐treated patients compared with non‐cancer controls. Data are presented as mean ± SEM (*N* = 3 patients per group). Individual dots (●) represent individual ROIs, with each color (grey, blue, and red) denoting a distinct patient within each group. Triangles (▲) represent the geometric mean of all ROIs across three brain sections per patient and are superimposed over their corresponding individual data points. P values were calculated using a nested *t*‐test.

### 3.9 T Cells Are Required for ICI‐Therapy‐Related Microglial Activation

Dysregulated glia, particularly microglial activation, is a hallmark of ICI‐related neurocognitive decline because microglial depletion using CSF1R inhibitors or genetic targeting has been shown to improve cognitive decline [18]. However, the sequence of events upstream of microglial activation, particularly the role of T cells, which are primary targets of ICI [36, 37], is not well established. Therefore, we examined the extent to which T cells mediate ICI‐therapy‐related microglial activation independent of tumor burden. To robustly delete T cells, we used a transgenic mouse model harboring a Cre‐inducible diphtheria toxin receptor (iDTR) transgene (C57BL/6‐Gt(ROSA)26Sortm1(HBEGF)Awai/J; B6‐iDTR). The cross of B6‐iDTR mice with CD4‐Cre transgenic mice results in F1 offspring mice (CD4‐Cre^±^ DTR^+/−^ or T‐DTR). T‐DTR mice enable selective depletion of T lymphocytes following diphtheria toxin (DT) administration because both CD4^+^ and CD8^+^ T cells express Cre, as the CD4 transcriptional program is activated during the double‐positive stage of thymic development. First, we validated T cell depletion by flow cytometry, which showed approximately 95% T cell depletion in the spleen 3 days after DT exposure (Figure S10).

To investigate the T cell–microglia inflammatory axis, ICI treatments were administered for three weeks, intraperitoneally, as described above. T cells were depleted either 2 days before or 7 days after initiation of ICI treatment, and brains were collected 72 h after the last ICI injection to assess microglial activation using dual (CD68‐IBA1) immunofluorescence staining (Figure 8A). CD68 is a lysosomal membrane protein and an indicator of phagocytosis/activation, and IBA1 is a pan‐microglial marker. Because T cells are essential for ICI‐mediated antitumor responses, we did not induce tumors in T cell depletion experiments. WT mice (C57Bl/6) receiving vehicle or ICI treatment were used for comparisons. ICI treatment of WT mice showed a significant elevation in the CD68^+^ and IBA1^+^ activated microglia compared with vehicle‐treated WT mice (*P* < 0.05, Figure 8B–D; Figure S5). In contrast, T cell depletion in T‐DTR mice at 2 days before or 7 days after ICI treatment did not elevate microglial activation, and the CD68‐IBA1 dual‐immunoreactivity was comparable to that of WT mice receiving vehicle. DT‐ and vehicle‐treated T‐DTR mice (without T cell depletion) showed no changes in CD68‐IBA1 immunoreactivity, indicating that DT or T cell depletion during homeostasis does not affect the microglial activation state. Together, these data show that T cells are required for ICI‐related microglial dysregulation and support the working model that ICI‐unleashed T cells are the key drivers of microglial activation during ICI therapy.

**FIGURE 8:**
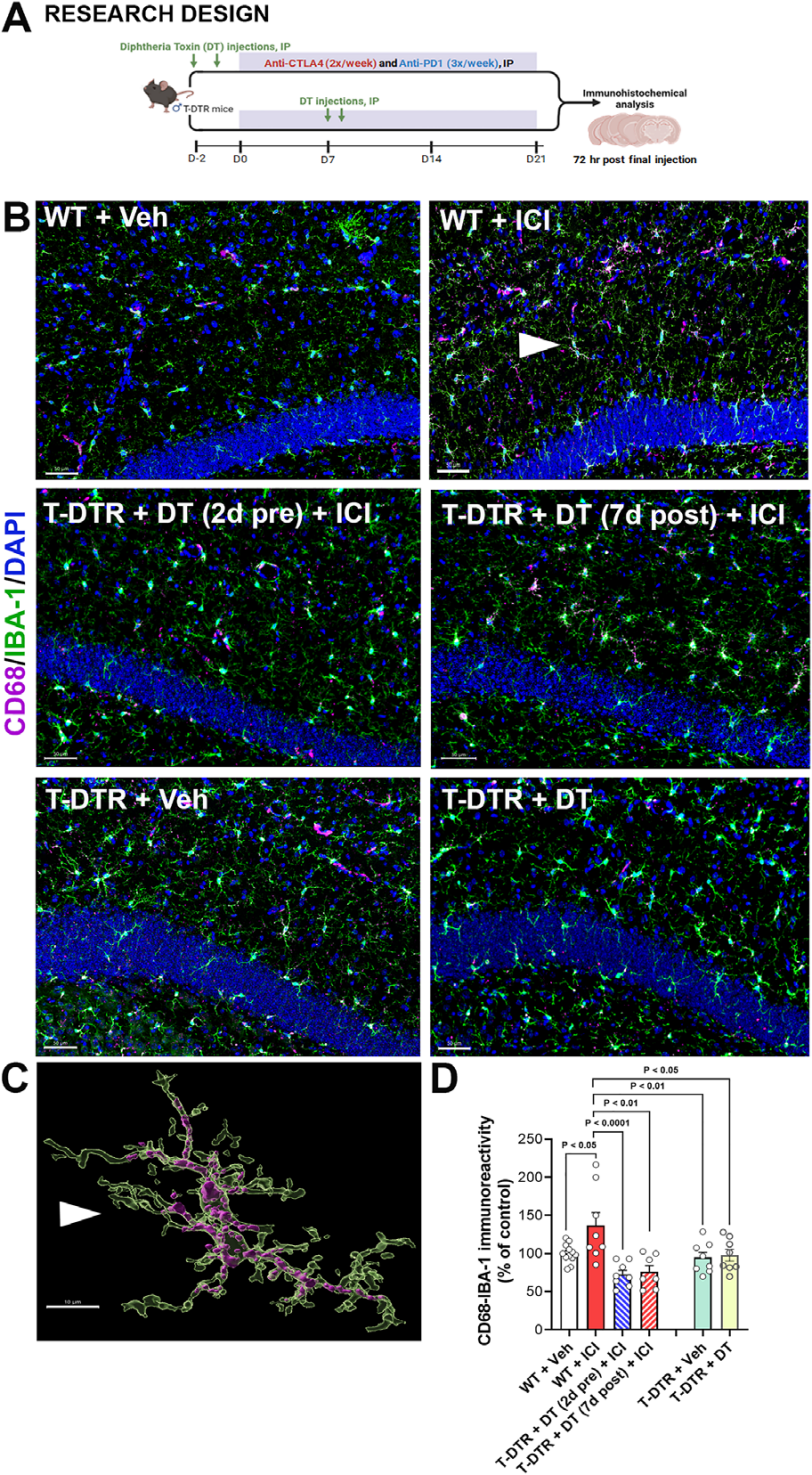
Depletion of T cells prevents ICI treatment‐induced microglial activation. (A) Experimental Design: Transgenic T‐DTR mice received diphtheria toxin A (DT) either 2 days before or 7 days after ICI treatment (anti‐CTLA‐4 and anti‐PD‐1). ICI treatments were given for 3 weeks (see Materials & Methods for details). WT (C57Bl/6) mice receiving either ITC or ICI treatment were used for comparison. Brains were collected at 72 h after completion of ICI treatments. (B) Representative dual‐immunofluorescence confocal z‐stacks depicting microglial activation (CD68, magenta, IBA1, green, and DAPI nuclear counterstaining) from T‐DTR and WT mouse hippocampus. (C) A 3D surface rendering of CD68‐IBA1^+^ activated microglia from the WT + ICI group (white arrows). (D) Volumetric quantification of CD68 and IBA1 immunoreactivity showing ICI treatment elevated microglial activation in WT + ICI group compared to WT + Vehicle. T cell depletion (T‐DTR mice) did not lead to microglial activation. Data are presented as Mean ± SEM, *N *= 6–8 mice/group. *P*‐values were derived from ANOVA and Dunn's multiple comparisons test. Scale bar, 50 µm (A,B).

## 4 Discussion

Our study reports a spatial transcriptomic profile of cell–type–specific markers and their associated regulatory networks following Combi‐ICI therapy. We identify an altered neuroimmune landscape, including inflammatory microglial activation, reactive astrogliosis, reduced myelin integrity in oligodendrocytes, and an NF‐κB‐associated signature in T cells. Notably, immunofluorescence analysis of postmortem brains from patients treated with anti‐PD‐1 therapy revealed microglial activation and reduced myelin, paralleling observations in our animal models. Mechanistically, complementary evidence from T cell‐depleting experiments demonstrates that ICI‐driven microglial activation depends on T cells. Collectively, our findings not only reveal cellular and molecular networks that underlie irAEs in the brain but also establish T cells as the key drivers of microglial dysregulation during ICI therapy.

### 4.1 Hippocampal RNA‐Seq Reveals Coordinated T Cell, Microglial, and Astrocyte Activation Signatures

Using a syngeneic non‐metastatic melanoma mouse model treated with a combination of anti‐CTLA‐4 and anti‐PD‐1, we previously identified selective impairment in hippocampal‐dependent cognitive function [16]. Building on these findings and to minimize the biological variability in our spatial transcriptomic analyses, we used adult male C57BL/6 mice, injected with D4M‐3A.UV2 melanoma cells. This parental D4M.3.A line was originally derived from male transgenic mice, limiting us to males to avoid potential tumor rejection in females (Y‐antigen response). Our results align with observed behavioral outcomes of ICI in pre‐clinical models [16, 18] and further address the spatial context of neuro‐inflammatory signaling, particularly microglial activation. Additionally, based on our previous preclinical findings, we selected the early time point to capture the increased microglial activation following ICI treatment.  Clinical reports showing cognitive symptoms in patients after ICI treatment [9], further supports the potential relevance of early ICI‐associated neuroinflammatory sequelae in contributing to cognitive vulnerability.

Bulk RNA sequencing of the hippocampus revealed robust upregulation of microglial‐, astrocytic‐, and T cell‐associated neuroinflammatory markers in Control + ICI vs Melanoma + ICI group, indicating a sustained inflammatory response following ICI therapy. Among the significantly upregulated genes common to both ICI‐treated groups, *Adora2a* (adenosine A2A receptor) emerged as a key candidate linked to neuromodulatory signaling. Notably, A2AR is expressed in astrocytic processes and microglia in the hippocampus [38], which is known to enhance neuronal excitability and can promote neuronal injury and cell death [39, 40]. Importantly, this overexpression has been linked to neuroinflammation and memory impairment [41]. Consistent with these findings in our dataset, *Adora2a* expression was elevated in Melanoma + ICI, supporting a notion that the adenosine–A2A axis has a potential role in astrocytic dysfunction and neuroinflammation following ICI treatment.

Furthermore, the top DEGs (*p* < 0.05) across datasets were identified, including microglia‐ and synapse‐related markers that were upregulated in both the Melanoma and Melanoma + ICI groups. Prior studies have shown that impaired MHC class II expression can lead to profound immune perturbations associated with CD4^+^ T cells [42]. *H2‐Eb1, H2‐Ab1, and H2‐A*a encode the β and α chains of murine MHC class II (I‐A/I‐E) and are canonical markers of MHC II‐high activated microglia/macrophages in the CNS [43]. Upregulation of these genes in myeloid‐origin cells is typically interpreted as enhanced antigen‐presenting capacity, which promotes CD4^+^ T cell priming and supports anti‐PD‐1/anti‐CTLA‐4 responses in brain metastases [44]. Similarly, the upregulation of *Darp32 (*a neural signaling and plasticity‐related gene) in the Melanoma + ICI group suggests a potential link between altered dopaminergic signaling [45] and cognitive impairment following ICI therapy. Together, these findings suggest inflammatory signatures post‐ICI therapy.

Evaluation of transcriptomic signatures across datasets further identified several strongly upregulated genes, including *Tmem252, Lcn2, Apold1, Rarb, Ptprv, Adora2a, and Cd4*, in the Melanoma + ICI group. Consistent with earlier studies, higher *CD4* expression was reported in responders within the ipilimumab‐treated subgroup [46], supporting a role for immune activation in the context of ICI therapy. In our experimental dataset, ICI treatment significantly increased *Ptprv* expression in both Melanoma and Melanoma + ICI groups. PTPRV, protein tyrosine phosphatase receptor V, is one of the downstream targets of p53 [24]. The p53 transcriptional activity has also been shown to modulate microglial pro‐inflammatory functions while suppressing anti‐inflammatory and tissue repair responses [47]. Collectively, these findings suggest that the p53‐PTPRV potentially contributes to microglial activation and neuroinflammation after ICI therapy.

### 4.2 Cross‐Species Comparisons of Cell Type‐Specific Spatial Gene Signatures Reveal ICI‐Linked Neuroinflammation Across Glia and T Cells

Disturbance in CNS homeostasis (e.g., neuroinflammation) triggers microglial activation with morphological changes and increased inflammatory signaling [48]. Moreover, oligodendrocytes also actively participate by interacting with microglia, astrocytes, and infiltrating or tissue‐resident immune cells, altering their signaling programs and releasing pro‐inflammatory cytokines [49]. Together, these observations emphasize that neuroinflammation is network‐driven, arising from coordinated crosstalk among microglia, astrocytes, and oligodendrocytes. However, how these interactions among non‐neuronal cells and inflammatory immune cells are altered by ICI therapy was largely undefined. Our integrated and targeted MERFISH approach provides new insights into markers across oligodendrocytes, astrocytes, T cells, and microglia, emphasizing the need to (i) delineate multi‐cellular interactions, (ii) gain a better understanding of ICI‐associated neuroinflammatory and neurodegenerative sequelae, and (iii) guide safer therapeutic strategies. Analysis of nearest‐neighbor spatial proximity revealed heterogeneous, relationship‐specific changes among T cells, microglia, astrocytes, and oligodendrocytes. A distance threshold of 50 µm was chosen to identify broader spatial proximity between source–target cell pairs [50]. T cell–microglia proximity was increased or had a shorter median distance in the Control + ICI and Melanoma + ICI groups, suggesting that ICI treatment may enhance local neuroimmune spatial association in the melanoma‐bearing brain. The reduction in median nearest‐neighbor distance between T cells and microglia in the Melanoma + ICI group is consistent with a prior spatial analysis report showing that increased T cells and macrophages form functionally relevant neighborhoods, and that their relative positioning is associated with immunotherapy response [51]. Moreover, studies have shown that microglia transcriptomics reprogramming toward a proinflammatory state is associated with better responses to ICI in mice and patients [52].

Similarly, T cell proximity to neighboring glial populations, including astrocytes, showed more prominent (shorter median distance) proximity in the Melanoma + ICI group. IrAEs induced by ICI therapy arise primarily from a loss of immune tolerance [53]. Although these effects are largely attributed to increased T cell activity, resident glial cells, including astrocytes and microglia, may further amplify the neuroinflammatory response. Furthermore, relatively short microglia–astrocyte and microglia–oligodendrocyte distances were observed in the Melanoma + ICI and Control + ICI groups, respectively. These findings may reflect enhanced spatial association of activated microglia (*Aif1^+^* and *Fcgr3^+^*) with neighboring glial populations in response to ICI‐associated neuroinflammation, including closer localization to oligodendrocyte and myelin compartments. Previous studies have shown that immune checkpoint blockade can promote interferon‐responsive microglial and oligodendrocyte states, leading to loss of oligodendrocytes and hypomyelination [54]. Likewise, we observed reduced myelin basic protein expression in white matter of ICI‐treated cancer and non‐cancer mouse brains [16]. Thus, the reduced distances observed in the ICI‐treated groups are consistent with increased microglia–glia crosstalk, although spatial proximity alone does not demonstrate direct cellular interaction. Additional functional studies will be needed to define the underlying mechanisms and biological consequences of these spatial proximity changes.

Gene expression changes based on unbiased cell clustering showed that ICI therapy drives microglia toward activated and immune‐regulatory states. In the Control + ICI group, microglial clusters showed elevated *Aif1* and *Fcgr3*, supporting a shift toward an activated phenotype. This pattern is consistent with prior studies identifying *Aif1* as a dynamically regulated injury‐associated marker involved in neuronal damage [55], and the association of *Fcgr3a* with immune signaling and altered responses to anti‐tumor therapies [55]. Importantly, our concurrent analysis in postmortem human brains showed elevated Fcgr3/CD16 expression in anti‐PD‐1‐treated patients compared to noncancer controls, further supporting an inflammatory microglial signature following ICI therapy.

Complementing these observations, our gene‐level analysis showed that *Gpr84* (a microglia‐associated pro‐inflammatory GPCR) was significantly upregulated in both the Melanoma and Melanoma + ICI groups. Mechanistically, increased *Gpr84* expression in microglia has been linked to NLRP3 inflammasome activation, potentially through inhibition of the cAMP/PKA signaling pathway [56]. Collectively, these results indicate that Combi‐ICI elicits a heterogeneous microglial response, driving microglia toward inflammatory and immune‐regulatory states (*Aif1/Fcgr3/Cd300lf*) and inducing sustained *Gpr84* signaling, suggesting an elevated neuroinflammatory state following ICI therapy.

Our spatial transcriptomic profiling of T cell markers revealed a distinct shift in T cell programs in response to melanoma and ICI therapy. In the Melanoma and Melanoma + ICI groups, we observed reduced expression of *Cd3e, Cd28, and Cd8a*, key genes involved in T cell development, activation, and differentiation. In contrast, the Melanoma + ICI group showed a higher proportion of cells expressing *Nfkbiz*. NF‐κB (including the gene variants *Nfkb1 and Nfkbiz*) is a transcription factor that plays a central role in neuroinflammation. Increased NF‐κB activity, often reflected by elevated pro‐inflammatory cytokine expression, can contribute to neuronal degeneration [57]. These prior findings corroborate our spatial transcriptomic analysis of T ell markers, which reveals enhanced NF‐κB–associated inflammatory signaling following ICI therapy [31]. However, further validation in ICI‐treated samples using T‐cell markers such as CD4 and CD8 will strengthen the cross‐species relevance of our transcriptomic data. Together, our spatial profiling indicates that post‐ICI T cells display an NF‐κB–associated inflammatory activation signature (increased *Nfkbiz/Nfkb1*) despite reduced expression of canonical activation/cytotoxic markers (decreased *Cd3e/Cd28/Cd8a*).

Neuroinflammatory responses involves coordinated signaling among multiple glial populations, and we detected astrocyte‐associated changes following ICI treatment. Across astrocyte clusters, a large proportion of cells expressed *Gja1 and Gfap*, with the strongest *Gfap*‐associated reactivity observed in the Melanoma and Melanoma + ICI groups. *Gja1*, also known as connexin 43 (*Cx43*), is a member of the connexin family of gap junction proteins [58]. Clinical observations in ICI‐treated patients have reported anti‐GFAP autoantibodies in cerebrospinal fluid (CSF), implicating astrocyte‐directed immune responses in immune‐related adverse events [59, 60]. Consistent with these reports, our gene‐level analyses also showed *Gfap* upregulation in ICI‐treated experimental groups, suggesting that Combi‐ICI‐associated astrocyte changes are driven by reactive‐state programs. Importantly, when translated into a human context, anti‐PD‐1‐treated patient brains showed dysregulated astrocyte markers compared with noncancer controls, including significantly increased *Gfap* and *Serpina3* immunoreactivity. Together, these cross‐species data support the conclusion that ICI is associated with heightened *Gfap*‐linked astrocyte reactivity, indicating that ICI‐related astrocyte responses are dominated by a reactive inflammatory state rather than restoration of astrocyte homeostasis.

Furthermore, we experimentally validated that ICI treatment disrupt oligodendrocyte homeostasis and myelin‐associated programs, supporting a broader role for glial dysregulation in ICI‐associated neurotoxicity. Oligodendrocyte sub‐clustering indicated that Combi‐ICI therapy alone exerts a comparatively modest effect on core oligodendrocyte and myelin programs, whereas melanoma is associated with more pronounced dysregulation. In particular, *Unc5b* expression was reduced in Melanoma and Melanoma + ICI. This observation is supported by prior reports showing that *Unc5b* is enriched in mature oligodendrocytes and is required for myelin sheath stability [61], supporting the interpretation that tumor‐bearing conditions are linked to compromised oligodendrocyte homeostasis and myelin maintenance. Additionally, the elevated *Sox10* in the ICI group is consistent with prior reports linking *Sox10* to immune checkpoint regulation: Yokoyama and colleagues reported that *Sox10* suppresses PD‐L1 expression via the IRF4–IRF1 axis [62], whereas Sasaki et al. reported increased PD‐L1 expression following *Sox10* overexpression [63]. Further studies are warranted to clarify the relationship between *Sox10* and immune checkpoint signaling. Translationally, anti‐PD‐1‐treated patient brains also showed elevated SOX10 immunoreactivity, which differs from patterns observed in the mouse oligodendrocyte compartment and may reflect differences in disease stage, region, or cellular composition across species. Importantly, the reduction of MBP immunoreactivity in ICI‐treated patient brains aligns with the myelin‐instability signature suggested by reduced *Unc5b* in the mouse dataset. Collectively, these findings not only corroborate our earlier observation of reduced MBP immunoreactivity [16] but also define a consistent oligodendrocyte/myelin dysregulation signature, encompassing *Unc5b* downregulation, *Sox10* perturbation, and MBP loss, supporting the model that dysregulated glia are at the center of ICI treatment‐induced neurotoxicity.

### 4.3 ICI‐Driven Microglial Activation Requires T Cells

ICIs enhance anti‐tumor immunity by blocking the inhibitory CTLA‐4 and PD‐1 signaling, thereby unleashing peripheral and tissue‐resident T‐cell responses. Although this mechanism is therapeutically beneficial against cancer, it can also disrupt neuroimmune homeostasis in the CNS. Multi‐omic studies in patients with CNS irAEs have identified infiltrating proinflammatory memory T‐helper cells associated with IL‐17 signaling, while inhibition of IL‐17 improved clinical outcomes [64]. Based on these observations, we conceptualize that ICI unleashes T cells while concurrently disrupting the PD1–PDL1 axis in the brain, thereby activating microglia and amplifying local neurodegenerative inflammatory loops. Our spatial transcriptomic profiling supports this concept based on coordinated alterations in both T cells and microglia following ICI exposure. Consistent with these transcriptional changes, spatial proximity analysis showed reduced nearest‐neighbor distances between T cells and microglia in the Melanoma + ICI group, suggesting that ICI treatment may enhance local neuroimmune interactions between T cells and microglia. In our study, conditional T cell depletion in transgenic T‐DTR mice prevented ICI‐induced microglial activation, as CD68/IBA1 dual immunoreactivity remained low and comparable to WT vehicle‐treated controls. These findings suggest that ICI first unleashes T cells, which subsequently drive microglial activation.

Our findings refine a model in which ICI treatment unleashes T cell activation that, in turn, drives secondary microglial reactivity in the brain. This interpretation is consistent with our previous studies showing increased numbers of IBA1^+^ microglial reactive cells in anti‐PD‐1‐treated mice [18]. It is also supported by a single‐cell RNA‐sequencing study showing that treatment with the engineered anti‐PD‐1 protein concurrently increased T cell abundance and microglial inflammation in the mouse brain [65]. Together with these observations, our transcriptomic findings, characterized by altered microglial homeostatic markers and increased expression of T cell activation markers, support a detrimental T cell–microglia inflammatory axis. T‐DTR depletion experiments provide functional validation of this axis, demonstrating that elimination of T cells attenuates ICI‐induced microglial activation and identifying T cells as key upstream drivers of microglial reactivity. These results suggest that limiting T cell‐mediated neuroinflammation may represent a protective strategy against ICI‐treatment‐induced neurodegenerative consequences.

One limitation of our study is that we focused primarily on changes in gene expression and cellular composition in the hippocampus related to cognitive impairment following Combi‐ICI therapy. However, future studies investigating interactions between non‐neuronal cells and inflammatory immune cells in other brain regions, particularly the prefrontal cortex, choroid plexus, and meninges, are warranted to better understand how these crosstalk networks contribute to neuroinflammation and cognitive dysfunction after ICI treatment. Availability of untreated melanoma patients or ICI‐exposed non‐cancer human tissues is clinically scarce. Thus, our human cohort compares non‐cancer controls with ICI‐treated cancer patients, with an inherent limitation in distinguishing ICI‐specific effects from cancer‐associated inflammation, systemic disease burden, or prior therapies. In addition, spatial transcriptomic profiling of brain tissue from patients receiving ICI therapy is needed, as it remains unclear to what extent the cell‐type‐specific markers identified in our experimental mouse model are conserved in the human brain. Related to the T cell–microglia axis, T cell depletion efficiency was estimated in the spleen (periphery); it remains unclear how much this reflects elimination of T cells in the brain and meninges. Thus, future studies could address the specific role of CNS‐associated T cells in driving microglial activation in response to ICI. Furthermore, the underlying molecular mechanisms of T cell–microglial crosstalk, including key immune signaling pathways such as IFNγ and TNFα, remain to be elucidated. Additional investigations are needed to determine the extent to which T cell depletion can attenuate or reverse ICI‐associated cognitive dysfunction.

## 5 Conclusion

In summary, our work defines cellular and molecular networks that drive neuroinflammation following ICI therapy. Spatial transcriptomic profiling of the mouse brain revealed cell type‐specific transcriptomic alterations indicative of neuroinflammatory dysregulation and disrupted glial homeostasis following ICI therapy. These findings were further supported by the human postmortem brain data, where upregulation of key neuroinflammatory markers, including Cd16/Fcgr3, Aif1, Gfap, and Sox10, coincided with reduced expression of myelin stability markers and elevated microglial activation. Collectively, our results support that altered intercellular crosstalk among microglia, astrocytes, and oligodendrocytes represents a pathophysiological hallmark of ICI‐associated neuroinflammation. In addition, our findings clearly demonstrate that T cells are essential players in ICI‐driven microglial activation. Our study not only establishes a robust mechanistic framework for how ICI therapy alters neuroinflammatory pathways but also provides opportunities to develop strategies to decouple microglial activation from the anti‐tumor effects of ICI therapy.

## Author Contributions

M.M.A. and S.O. conceptualized, designed, and supervised this study. D.S. and S.M.E.K. developed methodologies and conducted data acquisition. E.N. established the T cell depletion model. D.S., S.M.E.K., S.S., S.O., & M.M.A. carried out the analysis and interpretation of data. J.M.V., R.Z., S.O., and M.M.A. provided administrative, technical, or material support. D.S., J.M.V., R.Z., S.O., & M.M.A conducted writing, reviewing, and/or revising the manuscript.

## Funding

This work was supported by the National Institutes of Health (NIH) awards (R01CA251110, R01CA262213) and the UC Irvine Chao Family Comprehensive Cancer Center (CFCCC) Pilot Award to M.M.A. NIH R01AI168063 and R21AI196939 awards to S.O. UCI CFCCC and GRTH were supported by the NIH program project award P30CA062203.

## Ethics Statement

All animal experiments were approved by the UCI Institutional Animal Care and Use Committee (IACUC). All human brain tissue experiements were approved by the institutional ethics committees.

## Conflicts of Interest

The authors declare no conflicts of interest.

## Supporting information




**Supporting File**: advs77733‐sup‐0001‐SuppMat.docx.

## Data Availability

The data that support the findings of this study are available from the corresponding author upon reasonable request.The MERFISH data in this study were deposited in the Gene Expression Omnibus (GEO) repository under accession code GSE322277. The bulk RNA seq data were deposited in the GEO repository under accession number GSE322681.
