# Supplementary material for: Spatial Transcriptomics Reveals a T Cell‐Mediated Microglial Activation Axis of Immune Checkpoint Inhibition‐Related Adverse Events in the Brain
Source: Adv Sci (Weinh). 2026 Sep 30:e77733. Online ahead of print. doi: 10.1002/advs.77733 (PMC13624869; doi:10.1002/advs.77733)
Supplement: Supplementary file 1 [file ADVS-9999-e77733-s001.docx]

**Suppl. Figure S1**

**A**

Center_Y

Center_Y

Center_X

Center_X

**B**

**Supplemental Figure S1. ROI-based non-neuronal cell distribution across hippocampal subregions.**

**(A)** Hippocampal subregions from sections of experimental group were identified by manually drawing polygonal ROIs on the spatial coordinate map using center_x and center_y values. ROIs were created for CA1, CA3, DG, and Subiculum according to their anatomical locations in the section. Cells falling within each polygon were assigned to the corresponding hippocampal region and used for downstream analysis of regional non-neuronal cell distribution

**(B)** MERFISH-profiled cells were assigned to CA1, CA3, DG, and Subiculum using ROI-based annotation, and non-neuronal cell classes were identified by cluster-level marker matching. Stacked horizontal bar plots illustrate the number of cells in each non-neuronal class across regions

**Suppl. Figure S2**

**Supplemental Figure S2.** Heatmaps and corresponding enriched pathways showing fold changes and expression patterns of upregulated genes across Melanoma, and Melanoma ICI groups

**Supplementary Figure S3**

.

**Supplemental Figure S3. Spatial cell type maps across all datasets.** Heatmap showing the relative percentage of predicted cell types across Control, Control + ICI, Melanoma and Melanoma + ICI

**Suppl. Figure S4**

Control	Control +ICI	Melanoma	. Melanoma + ICI

**Supplemental Figure S4.** ROI-based spatial T cell-Microglia proximity box plot across hippocampal subregions.

**Suppl. Figure S5**

**A**

**Supplemental Figure S5. A. Neighborhood proximity analysis of T cell–microglia pairs with astrocytes and oligodendrocytes.** Heat map showing the median Euclidean nearest-neighbor distance (50 um) for T cell–astrocyte, T cell–oligodendrocyte, microglia–astrocyte, and microglia–oligodendrocyte spatial relationships within the hippocampal region. Lower median distances indicate closer spatial proximity or greater interaction, whereas higher distances indicate greater spatial separation.

**Suppl. Figure S5 (contd.)**

 **B	T cell- nearest Astrocytes	T cell- nearest Oligodendrocytes**

**CONTROL**

**CONTROL + ICI**

**MELANOMA**

**MELANOMA + ICI**

T cells

Oligo

Astrocytes

**Suppl. Figure S5 (contd.)**

**C	Microglia-nearest Astrocytes	Microglia-nearest Oligodendrocytes**

**CONTROL**

**CONTROL + ICI**

**MELANOMA**

**MELANOMA + ICI**

Microglia

Oligo

Astrocytes

**Supplemental Figure S5**. **(B)** Spatial mapping and nearest-neighbor proximity analysis of T cells with nearest astrocytes, and oligodendrocytes **(C)** Microglia with nearest astrocytes, and oligodendrocytes across treatment groups. Cell types were assigned using log-transformed marker-expression scores and conservative marker-detection thresholds. Nearest-neighbor analysis was used to calculate pairwise spatial distances between cell populations.

**Suppl. Figure S6**

**A**

**B**

.

**Supplemental Figure S6**. **Microglial subcluster composition and marker gene expression profiling across experimental groups.**

**(A)** Microglia subcluster were identified using canonical marker genes and re-clustered using Seurat graph-based clustering generating sub clusters relabeled as M1-M16. The proportion of cells assigned to each Microglia cluster across datasets was summarized using 100% stacked bar plots

**(B)** Heat map showing average gene expression of selected Microglia marker genes. Expression values were scaled row-wise and visualized as Z-scores to highlight relative differences across clusters.

**Suppl. Figure S7**

**A**

**B**

**Supplemental Figure S7**. **T cell subcluster composition and marker gene expression profiling across experimental groups.**

**(A)** T cells subcluster were identified using canonical marker genes and re-clustered using Seurat graph-based clustering generating sub clusters relabeled as T1-T16. The proportion of cells assigned to each T cell cluster across datasets was summarized using 100% stacked bar plots

**(B)** Heat map showing average gene expression of selected T cell marker genes. Expression values were scaled row-wise and visualized as Z-scores to highlight relative differences across clusters.

**Suppl. Figure S8**

**A**

**B**

**Supplemental Figure S8**. **Astrocytes subcluster composition and marker gene expression profiling across experimental groups.**

**(A)** Astrocytes subcluster were identified using canonical marker genes and re-clustered using Seurat graph-based clustering generating sub clusters relabeled as A1-A18. The proportion of cells assigned to each Astrocyte cluster across datasets was summarized using 100% stacked bar plots

**(B)** Heat map showing average gene expression of selected Astrocyte marker genes. Expression values were scaled row-wise and visualized as Z-scores to highlight relative differences across clusters.

**Suppl. Figure S9**

**A**

**B**

**Supplemental Figure S9**. **Oligodendrocytes subcluster composition and marker gene expression profiling across experimental groups.**

**(A)** Oligodendrocytes subcluster were identified using canonical marker genes and re-clustered using Seurat graph-based clustering generating sub clusters relabeled as O1-O18. The proportion of cells assigned to each Oligodendrocyte cluster across datasets was summarized using 100% stacked bar plots

**(B)** Heat map showing average gene expression of selected Oligodendrocyte marker genes. Expression values were scaled row-wise and visualized as Z-scores to highlight relative differences across clusters.

**Suppl. Figure S10**

**A**	***PBS***

TCRβ

FSC-H

SSC-H

FSC-H

FSC-A

NK1.1

All cells

Single cells

T cells

CD45^+^ cells

Non NKT

NK cells

CD45

FSC-A

***DT***

T cells

Non NKT

NK cells

CD45^+^ cells

All cells

Single cells

FSC-H

SSC-H

FSC-H

FSC-A

NK1.1

CD45

FSC-A

TCRβ

| **Group** | **NK cells** | **Non-NKT**  **(B cells,**  **Myeloid cells)** | **T cells** |
| --- | --- | --- | --- |
| PBS | 2.44 × 10^6^ | 41.47 × 10^6^ | 11.35 × 10^6^ |
| DT | 2.63 × 10^6^ | 44.30 × 10^6^ | **0.73** × **x10^6^** |

**B**

**Supplemental Figure S10. T cell depletion efficacy.** T-DTR mice were administered diphtheria toxin (DT) or PBS via intraperitoneal route and spleens were collected on day 3 to verify T cell depletion by flow cytometry. (**A**) Example of gating strategy and representative flow plots (n=2) to identify immune (CD45^+^) cells, Natural killer (NK) cells (NK1.1^+^ TCRβ^-^), and T cells (NK1.1^-^ TCRβ^+^). (**B**) The number of splenic natural killer (NK), other immune cells and T cells, showcasing T cell depletion with **~95% efficiency**.

**Suppl. Table 1**

**Supplemental table 1:** Targeted Gene list for MERFISH panel encompassing cell gene with neuro-immune function

**Suppl. Table 2**

| **CONTROL** | **leiden** | **cell_count** | **total_cells_per_replicate** | **proportion** |
| --- | --- | --- | --- | --- |
| Replicate_1 | 0 | 364 | 6953 | 0.052351503 |
| Replicate_1 | 1 | 543 | 6953 | 0.078095786 |
| Replicate_1 | 2 | 857 | 6953 | 0.123256148 |
| Replicate_1 | 3 | 478 | 6953 | 0.068747303 |
| Replicate_1 | 4 | 684 | 6953 | 0.098374802 |
| Replicate_1 | 5 | 273 | 6953 | 0.039263627 |
| Replicate_1 | 6 | 309 | 6953 | 0.044441248 |
| Replicate_1 | 7 | 508 | 6953 | 0.073061988 |
| Replicate_1 | 8 | 355 | 6953 | 0.051057098 |
| Replicate_1 | 9 | 317 | 6953 | 0.045591831 |
| Replicate_1 | 10 | 331 | 6953 | 0.04760535 |
| Replicate_1 | 11 | 309 | 6953 | 0.044441248 |
| Replicate_1 | 12 | 349 | 6953 | 0.050194161 |
| Replicate_1 | 13 | 215 | 6953 | 0.030921904 |
| Replicate_1 | 14 | 242 | 6953 | 0.03480512 |
| Replicate_1 | 15 | 182 | 6953 | 0.026175751 |
| Replicate_1 | 16 | 182 | 6953 | 0.026175751 |
| Replicate_1 | 17 | 201 | 6953 | 0.028908385 |
| Replicate_1 | 18 | 108 | 6953 | 0.015532864 |
| Replicate_1 | 19 | 56 | 6953 | 0.008054077 |
| Replicate_1 | 20 | 63 | 6953 | 0.009060837 |
| Replicate_1 | 21 | 27 | 6953 | 0.003883216 |
| Replicate_3 | 0 | 113 | 1081 | 0.10453284 |
| Replicate_3 | 1 | 154 | 1081 | 0.142460685 |
| Replicate_3 | 2 | 32 | 1081 | 0.02960222 |
| Replicate_3 | 3 | 103 | 1081 | 0.095282146 |
| Replicate_3 | 4 | 29 | 1081 | 0.026827012 |
| Replicate_3 | 5 | 100 | 1081 | 0.092506938 |
| Replicate_3 | 6 | 61 | 1081 | 0.056429232 |
| Replicate_3 | 7 | 9 | 1081 | 0.008325624 |
| Replicate_3 | 8 | 88 | 1081 | 0.081406105 |
| Replicate_3 | 9 | 159 | 1081 | 0.147086031 |
| Replicate_3 | 10 | 16 | 1081 | 0.01480111 |
| Replicate_3 | 11 | 29 | 1081 | 0.026827012 |
| Replicate_3 | 12 | 1 | 1081 | 0.000925069 |
| Replicate_3 | 13 | 24 | 1081 | 0.022201665 |
| Replicate_3 | 14 | 10 | 1081 | 0.009250694 |
| Replicate_3 | 15 | 40 | 1081 | 0.037002775 |
| Replicate_3 | 16 | 20 | 1081 | 0.018501388 |
| Replicate_3 | 17 | 2 | 1081 | 0.001850139 |
| Replicate_3 | 18 | 18 | 1081 | 0.016651249 |
| Replicate_3 | 19 | 40 | 1081 | 0.037002775 |
| **CONTROL** | **leiden** | **cell_count** | **total_cells_per_replicate** | **proportion** |
| Replicate_3 | 20 | 25 | 1081 | 0.023126735 |
| Replicate_3 | 21 | 8 | 1081 | 0.007400555 |
| Replicate_2 | 0 | 598 | 2617 | 0.228505923 |
| Replicate_2 | 1 | 279 | 2617 | 0.106610623 |
| Replicate_2 | 2 | 26 | 2617 | 0.00993504 |
| Replicate_2 | 3 | 227 | 2617 | 0.086740543 |
| Replicate_2 | 4 | 47 | 2617 | 0.017959496 |
| Replicate_2 | 5 | 357 | 2617 | 0.136415743 |
| Replicate_2 | 6 | 306 | 2617 | 0.11692778 |
| Replicate_2 | 7 | 93 | 2617 | 0.035536874 |
| Replicate_2 | 8 | 121 | 2617 | 0.046236148 |
| Replicate_2 | 9 | 80 | 2617 | 0.030569354 |
| Replicate_2 | 10 | 80 | 2617 | 0.030569354 |
| Replicate_2 | 11 | 39 | 2617 | 0.01490256 |
| Replicate_2 | 12 | 5 | 2617 | 0.001910585 |
| Replicate_2 | 13 | 111 | 2617 | 0.042414979 |
| Replicate_2 | 14 | 82 | 2617 | 0.031333588 |
| Replicate_2 | 15 | 36 | 2617 | 0.013756209 |
| Replicate_2 | 16 | 16 | 2617 | 0.006113871 |
| Replicate_2 | 17 | 8 | 2617 | 0.003056935 |
| Replicate_2 | 18 | 35 | 2617 | 0.013374092 |
| Replicate_2 | 19 | 34 | 2617 | 0.012991976 |
| Replicate_2 | 20 | 25 | 2617 | 0.009552923 |

| **CONTROL + ICI** | **leiden** | **cell_count** | **total_cells_per_replicate** | **proportion** |
| --- | --- | --- | --- | --- |
| Replicate_1 | 0 | 37 | 1050 | 0.035238 |
| Replicate_1 | 1 | 80 | 1050 | 0.07619 |
| Replicate_1 | 2 | 35 | 1050 | 0.033333 |
| Replicate_1 | 3 | 101 | 1050 | 0.09619 |
| Replicate_1 | 4 | 16 | 1050 | 0.015238 |
| Replicate_1 | 5 | 112 | 1050 | 0.106667 |
| Replicate_1 | 6 | 94 | 1050 | 0.089524 |
| Replicate_1 | 7 | 92 | 1050 | 0.087619 |
| Replicate_1 | 8 | 77 | 1050 | 0.073333 |
| Replicate_1 | 9 | 96 | 1050 | 0.091429 |
| Replicate_1 | 10 | 32 | 1050 | 0.030476 |
| Replicate_1 | 11 | 32 | 1050 | 0.030476 |
| Replicate_1 | 12 | 46 | 1050 | 0.04381 |
| Replicate_1 | 13 | 13 | 1050 | 0.012381 |
| Replicate_1 | 14 | 41 | 1050 | 0.039048 |
| Replicate_1 | 15 | 32 | 1050 | 0.030476 |
| Replicate_1 | 16 | 21 | 1050 | 0.02 |
| **CONTROL + ICI** | **leiden** | **cell_count** | **total_cells_per_replicate** | **proportion** |
| Replicate_1 | 17 | 19 | 1050 | 0.018095 |
| Replicate_1 | 18 | 18 | 1050 | 0.017143 |
| Replicate_1 | 19 | 12 | 1050 | 0.011429 |
| Replicate_1 | 20 | 19 | 1050 | 0.018095 |
| Replicate_1 | 21 | 14 | 1050 | 0.013333 |
| Replicate_1 | 22 | 11 | 1050 | 0.010476 |
| Replicate_2 | 0 | 200 | 2114 | 0.094607 |
| Replicate_2 | 1 | 197 | 2114 | 0.093188 |
| Replicate_2 | 2 | 78 | 2114 | 0.036897 |
| Replicate_2 | 3 | 221 | 2114 | 0.104541 |
| Replicate_2 | 4 | 49 | 2114 | 0.023179 |
| Replicate_2 | 5 | 148 | 2114 | 0.070009 |
| Replicate_2 | 6 | 112 | 2114 | 0.05298 |
| Replicate_2 | 7 | 153 | 2114 | 0.072375 |
| Replicate_2 | 8 | 132 | 2114 | 0.062441 |
| Replicate_2 | 9 | 129 | 2114 | 0.061022 |
| Replicate_2 | 10 | 70 | 2114 | 0.033113 |
| Replicate_2 | 11 | 68 | 2114 | 0.032167 |
| Replicate_2 | 12 | 83 | 2114 | 0.039262 |
| Replicate_2 | 13 | 52 | 2114 | 0.024598 |
| Replicate_2 | 14 | 69 | 2114 | 0.03264 |
| Replicate_2 | 15 | 74 | 2114 | 0.035005 |
| Replicate_2 | 16 | 64 | 2114 | 0.030274 |
| Replicate_2 | 17 | 62 | 2114 | 0.029328 |
| Replicate_2 | 18 | 32 | 2114 | 0.015137 |
| Replicate_2 | 19 | 26 | 2114 | 0.012299 |
| Replicate_2 | 20 | 36 | 2114 | 0.017029 |
| Replicate_2 | 21 | 29 | 2114 | 0.013718 |
| Replicate_2 | 22 | 30 | 2114 | 0.014191 |
| Replicate_3 | 0 | 265 | 2209 | 0.119964 |
| Replicate_3 | 1 | 216 | 2209 | 0.097782 |
| Replicate_3 | 2 | 293 | 2209 | 0.132639 |
| Replicate_3 | 3 | 34 | 2209 | 0.015392 |
| Replicate_3 | 4 | 280 | 2209 | 0.126754 |
| Replicate_3 | 5 | 78 | 2209 | 0.03531 |
| Replicate_3 | 6 | 103 | 2209 | 0.046627 |
| Replicate_3 | 7 | 57 | 2209 | 0.025804 |
| Replicate_3 | 8 | 62 | 2209 | 0.028067 |
| Replicate_3 | 9 | 32 | 2209 | 0.014486 |
| Replicate_3 | 10 | 114 | 2209 | 0.051607 |
| Replicate_3 | 11 | 109 | 2209 | 0.049344 |
| Replicate_3 | 12 | 45 | 2209 | 0.020371 |
| **CONTROL + ICI** | **leiden** | **cell_count** | **total_cells_per_replicate** | **proportion** |
| Replicate_3 | 13 | 89 | 2209 | 0.04029 |
| Replicate_3 | 14 | 40 | 2209 | 0.018108 |
| Replicate_3 | 15 | 43 | 2209 | 0.019466 |
| Replicate_3 | 16 | 52 | 2209 | 0.02354 |
| Replicate_3 | 17 | 52 | 2209 | 0.02354 |
| Replicate_3 | 18 | 79 | 2209 | 0.035763 |
| Replicate_3 | 19 | 58 | 2209 | 0.026256 |
| Replicate_3 | 20 | 32 | 2209 | 0.014486 |
| Replicate_3 | 21 | 43 | 2209 | 0.019466 |
| Replicate_3 | 22 | 33 | 2209 | 0.014939 |

| **MELANOMA** | **leiden** | **cell_count** | **total_cells_per_replicate** | **proportion** |
| --- | --- | --- | --- | --- |
| Replicate_1 | 0 | 480 | 6068 | 0.079103 |
| Replicate_1 | 1 | 789 | 6068 | 0.130026 |
| Replicate_1 | 2 | 489 | 6068 | 0.080587 |
| Replicate_1 | 3 | 465 | 6068 | 0.076632 |
| Replicate_1 | 4 | 501 | 6068 | 0.082564 |
| Replicate_1 | 5 | 970 | 6068 | 0.159855 |
| Replicate_1 | 6 | 505 | 6068 | 0.083223 |
| Replicate_1 | 7 | 184 | 6068 | 0.030323 |
| Replicate_1 | 8 | 398 | 6068 | 0.06559 |
| Replicate_1 | 9 | 249 | 6068 | 0.041035 |
| Replicate_1 | 10 | 286 | 6068 | 0.047132 |
| Replicate_1 | 11 | 201 | 6068 | 0.033125 |
| Replicate_1 | 12 | 213 | 6068 | 0.035102 |
| Replicate_1 | 13 | 148 | 6068 | 0.02439 |
| Replicate_1 | 14 | 6 | 6068 | 0.000989 |
| Replicate_1 | 15 | 105 | 6068 | 0.017304 |
| Replicate_1 | 16 | 46 | 6068 | 0.007581 |
| Replicate_1 | 17 | 33 | 6068 | 0.005438 |
| Replicate_2 | 0 | 1256 | 6542 | 0.19199 |
| Replicate_2 | 1 | 430 | 6542 | 0.065729 |
| Replicate_2 | 2 | 719 | 6542 | 0.109905 |
| Replicate_2 | 3 | 708 | 6542 | 0.108224 |
| Replicate_2 | 4 | 580 | 6542 | 0.088658 |
| Replicate_2 | 5 | 65 | 6542 | 0.009936 |
| Replicate_2 | 6 | 340 | 6542 | 0.051972 |
| Replicate_2 | 7 | 601 | 6542 | 0.091868 |
| Replicate_2 | 8 | 297 | 6542 | 0.045399 |
| Replicate_2 | 9 | 321 | 6542 | 0.049068 |
| Replicate_2 | 10 | 178 | 6542 | 0.027209 |
| Replicate_2 | 11 | 245 | 6542 | 0.03745 |
| **MELANOMA** | **leiden** | **cell_count** | **total_cells_per_replicate** | **proportion** |
| Replicate_2 | 12 | 205 | 6542 | 0.031336 |
| Replicate_2 | 13 | 166 | 6542 | 0.025375 |
| Replicate_2 | 14 | 297 | 6542 | 0.045399 |
| Replicate_2 | 15 | 80 | 6542 | 0.012229 |
| Replicate_2 | 16 | 27 | 6542 | 0.004127 |
| Replicate_2 | 17 | 27 | 6542 | 0.004127 |

| **Melanoma + ICI** | **leiden** | **cell_count** | **total_cells_per_replicate** | **proportion** |
| --- | --- | --- | --- | --- |
| Replicate_1 | 0 | 895 | 5230 | 0.17112811 |
| Replicate_1 | 1 | 782 | 5230 | 0.14952199 |
| Replicate_1 | 2 | 328 | 5230 | 0.06271511 |
| Replicate_1 | 3 | 260 | 5230 | 0.04971319 |
| Replicate_1 | 4 | 202 | 5230 | 0.03862333 |
| Replicate_1 | 5 | 376 | 5230 | 0.07189293 |
| Replicate_1 | 6 | 271 | 5230 | 0.05181644 |
| Replicate_1 | 7 | 245 | 5230 | 0.04684512 |
| Replicate_1 | 8 | 251 | 5230 | 0.04799235 |
| Replicate_1 | 9 | 250 | 5230 | 0.04780115 |
| Replicate_1 | 10 | 260 | 5230 | 0.04971319 |
| Replicate_1 | 11 | 189 | 5230 | 0.03613767 |
| Replicate_1 | 12 | 151 | 5230 | 0.02887189 |
| Replicate_1 | 13 | 144 | 5230 | 0.02753346 |
| Replicate_1 | 14 | 173 | 5230 | 0.03307839 |
| Replicate_1 | 15 | 137 | 5230 | 0.02619503 |
| Replicate_1 | 16 | 151 | 5230 | 0.02887189 |
| Replicate_1 | 17 | 129 | 5230 | 0.02466539 |
| Replicate_1 | 18 | 36 | 5230 | 0.00688337 |
| Replicate_3 | 0 | 53 | 935 | 0.05668449 |
| Replicate_3 | 1 | 16 | 935 | 0.0171123 |
| Replicate_3 | 2 | 171 | 935 | 0.1828877 |
| Replicate_3 | 3 | 84 | 935 | 0.08983957 |
| Replicate_3 | 4 | 104 | 935 | 0.11122995 |
| Replicate_3 | 5 | 124 | 935 | 0.13262032 |
| Replicate_3 | 6 | 66 | 935 | 0.07058824 |
| Replicate_3 | 7 | 99 | 935 | 0.10588235 |
| Replicate_3 | 8 | 27 | 935 | 0.02887701 |
| Replicate_3 | 9 | 15 | 935 | 0.01604278 |
| Replicate_3 | 10 | 19 | 935 | 0.02032086 |
| Replicate_3 | 11 | 22 | 935 | 0.02352941 |
| Replicate_3 | 12 | 30 | 935 | 0.03208556 |
| Replicate_3 | 13 | 41 | 935 | 0.04385027 |
| Replicate_3 | 14 | 14 | 935 | 0.01497326 |
| Replicate_3 | 15 | 21 | 935 | 0.02245989 |
| Replicate_3 | 16 | 6 | 935 | 0.00641711 |
| Replicate_3 | 17 | 15 | 935 | 0.01604278 |
| Replicate_3 | 18 | 8 | 935 | 0.00855615 |
| Replicate_2 | 0 | 54 | 1338 | 0.04035874 |
| Replicate_2 | 1 | 14 | 1338 | 0.01046338 |
| Replicate_2 | 2 | 266 | 1338 | 0.19880419 |
| Replicate_2 | 3 | 236 | 1338 | 0.17638266 |
| Replicate_2 | 4 | 251 | 1338 | 0.18759342 |
| Replicate_2 | 5 | 49 | 1338 | 0.03662182 |
| Replicate_2 | 6 | 86 | 1338 | 0.06427504 |
| Replicate_2 | 7 | 34 | 1338 | 0.02541106 |
| Replicate_2 | 8 | 32 | 1338 | 0.02391629 |
| Replicate_2 | 9 | 38 | 1338 | 0.0284006 |
| Replicate_2 | 10 | 11 | 1338 | 0.00822123 |
| Replicate_2 | 11 | 32 | 1338 | 0.02391629 |
| Replicate_2 | 12 | 45 | 1338 | 0.03363229 |
| Replicate_2 | 13 | 38 | 1338 | 0.0284006 |
| Replicate_2 | 14 | 27 | 1338 | 0.02017937 |
| Replicate_2 | 15 | 47 | 1338 | 0.03512706 |
| Replicate_2 | 16 | 5 | 1338 | 0.00373692 |
| Replicate_2 | 17 | 2 | 1338 | 0.00149477 |
| Replicate_2 | 18 | 71 | 1338 | 0.05306428 |

**Supplemental table 2:** Biological replicate distribution across clusters : The table summarizes the distribution of cells across biological replicates for each identified cluster. For each experimental condition, cells were grouped by biological replicate and Leiden cluster. The cell counts and proportional cluster representation were calculated from each replicate

**Suppl. Table 3**

| **GROUP** | **No. of Patients** | **Gender** | **Age (median)** | **Therapy regime** | **Cause of Death** |
| --- | --- | --- | --- | --- | --- |
| ICI-Therapy | 3 | Female (0)  Male (3) | 65(59-73) | Pembrolizumab (1)  Nivolumab(1)  Ipilimumab/Nivolumab (1) | Lung cancer, melanoma, Head & neck cancer |
| Control | 3 | Female (1)  Male (2) | 59 (26-70) | Nil | Cardiogenic shock, thromboembolic event, hemorrhagic event, aortic dissection, multiple organ failure |

**Supplemental Table 3.** Human patient characteristics for the brain biopsy samples.

| **Dataset** | **celltype_comp** | **cell_count** | **proportion** | **% of cell** |
| --- | --- | --- | --- | --- |
| Control | Astrocytes | 341 | 0.032015773 | 3.20 |
| Control | Excitatory_Neurons | 6124 | 0.574969486 | 57.50 |
| Control | Microglia | 708 | 0.066472632 | 6.65 |
| Control | Oligodendrocytes | 1187 | 0.111444935 | **11.14** |
| Control | Synaptic function | 1862 | 0.174819266 | 17.48 |
| Control | T_cells | 57 | 0.00535161 | 0.54 |
| Control | T_cells & Complement-enriched | 372 | 0.034926298 | 3.49 |
| ControlICI | Astrocytes | 134 | 0.024939512 | 2.49 |
| ControlICI | Excitatory_Neurons | 3197 | 0.595012098 | 59.50 |
| ControlICI | Microglia | 444 | 0.082635399 | 8.26 |
| ControlICI | Oligodendrocytes | 343 | 0.063837707 | **6.38** |
| ControlICI | Synaptic function | 1102 | 0.205099572 | 20.51 |
| ControlICI | T_cells | 11 | 0.002047273 | 0.20 |
| ControlICI | T_cells & Complement-enriched | 142 | 0.026428438 | 2.64 |
| Melanoma | Astrocytes | 681 | 0.031023644 | 3.10 |
| Melanoma | Excitatory_Neurons | 7954 | 0.362352512 | 36.24 |
| Melanoma | Microglia | 1796 | 0.081818596 | 8.18 |
| Melanoma | Oligodendrocytes | 4215 | 0.192018587 | **19.20** |
| Melanoma | Synaptic function | 5746 | 0.26176484 | 26.18 |
| Melanoma | T_cells | 692 | 0.03152476 | 3.15 |
| Melanoma | T_cells & Complement-enriched | 867 | 0.039497062 | 3.95 |
| MelanomaICI | Astrocytes | 384 | 0.030452022 | 3.05 |
| MelanomaICI | Excitatory_Neurons | 6065 | 0.480967486 | 48.10 |
| MelanomaICI | Microglia | 848 | 0.067248216 | 6.72 |
| MelanomaICI | Oligodendrocytes | 2206 | 0.174940523 | **17.49** |
| MelanomaICI | Synaptic function | 2292 | 0.181760508 | 18.18 |
| MelanomaICI | T_cells | 310 | 0.024583664 | 2.46 |
| MelanomaICI | T_cells & Complement-enriched | 505 | 0.040047581 | 4.00 |

**Suppl. Table 4**

**Supplemental Table 4. Cluster based cell-type annotation**. Cell identities were assigned by comparing cluster marker genes with curated gene marker list and selecting the cell type with the highest marker overlap.

**Suppl. Table 5**

| **Dataset** | **Microglia_cluster** | **cell_count** | **proportion** | **% proportion** |
| --- | --- | --- | --- | --- |
| Control | M1 | 399 | 0.162 | 16.25 |
| ControlICI | M1 | 86 | 0.09 | 9.01 |
| Melanoma | M1 | 1707 | 0.204 | 20.36 |
| MelanomaICI | M1 | 1039 | 0.244 | 24.41 |
| Control | M2 | 495 | 0.202 | 20.15 |
| ControlICI | M2 | 113 | 0.118 | 11.83 |
| Melanoma | M2 | 1124 | 0.134 | 13.41 |
| MelanomaICI | M2 | 562 | 0.132 | 13.2 |
| Control | M3 | 299 | 0.122 | 12.17 |
| ControlICI | M3 | 249 | 0.261 | 26.07 |
| Melanoma | M3 | 1121 | 0.134 | 13.37 |
| MelanomaICI | M3 | 611 | 0.144 | 14.35 |
| Control | M4 | 312 | 0.127 | 12.7 |
| ControlICI | M4 | 88 | 0.092 | 9.21 |
| Melanoma | M4 | 552 | 0.066 | 6.58 |
| MelanomaICI | M4 | 353 | 0.083 | 8.29 |
| Control | M5 | 150 | 0.061 | 6.11 |
| ControlICI | M5 | 14 | 0.015 | 1.47 |
| Melanoma | M5 | 836 | 0.1 | 9.97 |
| MelanomaICI | M5 | 206 | 0.048 | 4.84 |
| Control | M6 | 192 | 0.078 | 7.82 |
| ControlICI | M6 | 82 | 0.086 | 8.59 |
| Melanoma | M6 | 526 | 0.063 | 6.27 |
| MelanomaICI | M6 | 354 | 0.083 | 8.32 |
| Control | M7 | 209 | 0.085 | 8.51 |
| ControlICI | M7 | 47 | 0.049 | 4.92 |
| Melanoma | M7 | 487 | 0.058 | 5.81 |
| MelanomaICI | M7 | 274 | 0.064 | 6.44 |
| Control | M8 | 38 | 0.015 | 1.55 |
| ControlICI | M8 | 5 | 0.005 | 0.52 |
| Melanoma | M8 | 546 | 0.065 | 6.51 |
| MelanomaICI | M8 | 282 | 0.066 | 6.62 |
| Control | M9 | 171 | 0.07 | 6.96 |
| ControlICI | M9 | 180 | 0.188 | 18.85 |
| Melanoma | M9 | 262 | 0.031 | 3.13 |
| MelanomaICI | M9 | 157 | 0.037 | 3.69 |
| Control | M10 | 49 | 0.02 | 2 |
| ControlICI | M10 | 16 | 0.017 | 1.68 |
| Melanoma | M10 | 473 | 0.056 | 5.64 |
| **Dataset** | **Microglia_cluster** | **cell_count** | **proportion** | **% Proportion** |
| Melanoma ICI | M10 | 109 | 0.026 | 2.56 |
| Control | M11 | 16 | 0.007 | 0.65 |
| ControlICI | M11 | 16 | 0.017 | 1.68 |
| Melanoma | M11 | 336 | 0.04 | 4.01 |
| MelanomaICI | M11 | 57 | 0.013 | 1.34 |
| Control | M12 | 50 | 0.02 | 2.04 |
| ControlICI | M12 | 10 | 0.01 | 1.05 |
| Melanoma | M12 | 195 | 0.023 | 2.33 |
| MelanomaICI | M12 | 120 | 0.028 | 2.82 |
| Control | M13 | 46 | 0.019 | 1.87 |
| ControlICI | M13 | 41 | 0.043 | 4.29 |
| Melanoma | M13 | 139 | 0.017 | 1.66 |
| MelanomaICI | M13 | 91 | 0.021 | 2.14 |
| Control | M14 | 13 | 0.005 | 0.53 |
| ControlICI | M14 | 4 | 0.004 | 0.42 |
| Melanoma | M14 | 46 | 0.005 | 0.55 |
| MelanomaICI | M14 | 27 | 0.006 | 0.63 |
| Control | M15 | 16 | 0.007 | 0.65 |
| ControlICI | M15 | 2 | 0.002 | 0.21 |
| Melanoma | M15 | 23 | 0.003 | 0.27 |
| MelanomaICI | M15 | 9 | 0.002 | 0.21 |
| Control | M16 | 1 | 0 | 0.04 |
| ControlICI | M16 | 2 | 0.002 | 0.21 |
| Melanoma | M16 | 10 | 0.001 | 0.12 |
| MelanomaICI | M16 | 6 | 0.001 | 0.14 |

**Supplemental Table 5. Relative distribution of Microglia subcluster across the datasets.**

Raw values corresponding to the stacked bar plots showing the relative proportions of microglial subclusters across the datasets. For each dataset, cells assigned to each cluster were counted and normalized to the total microglial cell number. Pairwise difference in cluster distribution between selected groups were evaluated using Fischer’s exact test, followed by Benjamini-Hochberg correction for multiple comparison. Significance level: adjusted P<0.05

**Suppl. Table 6**

| **Dataset** | **Tcell_cluster** | **cell_count** | **proportion** | **% proportion** |
| --- | --- | --- | --- | --- |
| Control | T1 | 769 | 0.098919475 | 9.89 |
| ControlICI | T1 | 166 | 0.046304045 | 4.63 |
| Melanoma | T1 | 3229 | 0.201459945 | 20.15 |
| MelanomaICI | T1 | 1671 | 0.172570484 | 17.26 |
| Control | T2 | 827 | 0.106380242 | 10.64 |
| ControlICI | T2 | 573 | 0.159832636 | 15.98 |
| Melanoma | T2 | 2285 | 0.142563015 | 14.26 |
| MelanomaICI | T2 | 1039 | 0.107301456 | 10.73 |
| Control | T3 | 931 | 0.119758168 | 11.98 |
| ControlICI | T3 | 236 | 0.065829847 | 6.58 |
| Melanoma | T3 | 1698 | 0.105939606 | 10.59 |
| MelanomaICI | T3 | 1343 | 0.138696685 | 13.87 |
| Control | T4 | 717 | 0.092230512 | 9.22 |
| ControlICI | T4 | 287 | 0.080055788 | 8.01 |
| Melanoma | T4 | 2011 | 0.125467931 | 12.55 |
| MelanomaICI | T4 | 1016 | 0.104926159 | 10.49 |
| Control | T5 | 788 | 0.101363519 | 10.14 |
| ControlICI | T5 | 364 | 0.10153417 | 10.15 |
| Melanoma | T5 | 1451 | 0.090529074 | 9.05 |
| MelanomaICI | T5 | 1001 | 0.103377053 | 10.34 |
| Control | T6 | 873 | 0.112297402 | 11.23 |
| ControlICI | T6 | 452 | 0.126080893 | 12.61 |
| Melanoma | T6 | 1109 | 0.069191415 | 6.92 |
| MelanomaICI | T6 | 1003 | 0.1035836 | 10.36 |
| Control | T7 | 1030 | 0.132492925 | 13.25 |
| ControlICI | T7 | 406 | 0.113249651 | 11.32 |
| Melanoma | T7 | 953 | 0.059458448 | 5.95 |
| MelanomaICI | T7 | 770 | 0.07952081 | 7.95 |
| Control | T8 | 832 | 0.107023411 | 10.7 |
| ControlICI | T8 | 619 | 0.172663877 | 17.27 |
| Melanoma | T8 | 762 | 0.047541802 | 4.75 |
| **Dataset** | **Tcell_cluster** | **cell_count** | **proportion** | **% proportion** |
| MelanomaICI | T8 | 550 | 0.056800578 | 5.68 |
| Control | T9 | 410 | 0.052739902 | 5.27 |
| ControlICI | T9 | 169 | 0.047140865 | 4.71 |
| Melanoma | T9 | 1091 | 0.06806838 | 6.81 |
| MelanomaICI | T9 | 586 | 0.060518434 | 6.05 |
| Control | T10 | 342 | 0.043992797 | 4.4 |
| ControlICI | T10 | 162 | 0.045188285 | 4.52 |
| Melanoma | T10 | 821 | 0.05122286 | 5.12 |
| MelanomaICI | T10 | 398 | 0.041102964 | 4.11 |
| Control | T11 | 153 | 0.019680988 | 1.97 |
| ControlICI | T11 | 68 | 0.018967922 | 1.9 |
| Melanoma | T11 | 377 | 0.023521338 | 2.35 |
| MelanomaICI | T11 | 192 | 0.019828566 | 1.98 |
| Control | T12 | 63 | 0.008103936 | 0.81 |
| ControlICI | T12 | 43 | 0.011994421 | 1.2 |
| Melanoma | T12 | 34 | 0.002121288 | 0.21 |
| MelanomaICI | T12 | 23 | 0.002375297 | 0.24 |
| Control | T13 | 10 | 0.001286339 | 0.13 |
| ControlICI | T13 | 5 | 0.0013947 | 0.14 |
| Melanoma | T13 | 106 | 0.006613427 | 0.66 |
| MelanomaICI | T13 | 33 | 0.003408035 | 0.34 |
| Control | T14 | 4 | 0.000514536 | 0.05 |
| ControlICI | T14 | 4 | 0.00111576 | 0.11 |
| Melanoma | T14 | 59 | 0.003681058 | 0.37 |
| MelanomaICI | T14 | 39 | 0.004027677 | 0.4 |
| Control | T15 | 11 | 0.001414973 | 0.14 |
| ControlICI | T15 | 17 | 0.00474198 | 0.47 |
| Melanoma | T15 | 35 | 0.002183679 | 0.22 |
| MelanomaICI | T15 | 17 | 0.001755654 | 0.18 |
| Control | T16 | 14 | 0.001800875 | 0.18 |
| ControlICI | T16 | 14 | 0.00390516 | 0.39 |
| Melanoma | T16 | 7 | 0.000436736 | 0.04 |
| **Dataset** | **Tcell_cluster** | **cell_count** | **proportion** | **% proportion** |
| MelanomaICI | T16 | 2 | 0.000206548 | 0.02 |

**Supplemental Table 6. Relative distribution of T cell subcluster across the datasets.**

Raw values corresponding to the stacked bar plots showing the relative proportions of T cell subclusters across the datasets. For each dataset, cells assigned to each cluster were counted and normalized to the total microglial cell number. Pairwise difference in cluster distribution between selected groups were evaluated using Fischer’s exact test, followed by Benjamini-Hochberg correction for multiple comparison. Significance level: adjusted P<0.05

**Suppl. Table 7**

| **Dataset** | **Astrocyte_cluster** | **cell_count** | **proportion** | **% Proportion** |
| --- | --- | --- | --- | --- |
| Control | A1 | 540 | 0.161097852 | 16.11 |
| ControlICI | A1 | 129 | 0.084534731 | 8.45 |
| Melanoma | A1 | 2171 | 0.215163528 | 21.52 |
| MelanomaICI | A1 | 1304 | 0.265905383 | 26.59 |
| Control | A2 | 631 | 0.188245823 | 18.82 |
| ControlICI | A2 | 220 | 0.144167759 | 14.42 |
| Melanoma | A2 | 1814 | 0.179781962 | 17.98 |
| MelanomaICI | A2 | 901 | 0.183727569 | 18.37 |
| Control | A3 | 586 | 0.174821002 | 17.48 |
| ControlICI | A3 | 444 | 0.29095675 | 29.1 |
| Melanoma | A3 | 776 | 0.07690783 | 7.69 |
| MelanomaICI | A3 | 409 | 0.083401305 | 8.34 |
| Control | A4 | 322 | 0.096062053 | 9.61 |
| ControlICI | A4 | 219 | 0.143512451 | 14.35 |
| Melanoma | A4 | 1174 | 0.116352825 | 11.64 |
| MelanomaICI | A4 | 493 | 0.100530179 | 10.05 |
| Control | A5 | 334 | 0.099642005 | 9.96 |
| ControlICI | A5 | 147 | 0.096330275 | 9.63 |
| Melanoma | A5 | 789 | 0.078196234 | 7.82 |
| MelanomaICI | A5 | 379 | 0.07728385 | 7.73 |
| Control | A6 | 153 | 0.045644391 | 4.56 |
| ControlICI | A6 | 25 | 0.0163827 | 1.64 |
| Melanoma | A6 | 980 | 0.097125867 | 9.71 |
| MelanomaICI | A6 | 233 | 0.047512235 | 4.75 |
| Control | A7 | 98 | 0.029236277 | 2.92 |
| ControlICI | A7 | 108 | 0.070773263 | 7.08 |
| Melanoma | A7 | 472 | 0.046778989 | 4.68 |
| MelanomaICI | A7 | 251 | 0.051182708 | 5.12 |
| Control | A8 | 38 | 0.011336516 | 1.13 |
| ControlICI | A8 | 3 | 0.001965924 | 0.2 |
| Melanoma | A8 | 547 | 0.054212091 | 5.42 |
| **Dataset** | **Astrocyte_cluster** | **cell_count** | **proportion** | **%proportion** |
| MelanomaICI | A8 | 280 | 0.057096248 | 5.71 |
| Control | A9 | 158 | 0.047136038 | 4.71 |
| ControlICI | A9 | 67 | 0.043905636 | 4.39 |
| Melanoma | A9 | 359 | 0.035579782 | 3.56 |
| MelanomaICI | A9 | 170 | 0.034665579 | 3.47 |
| Control | A10 | 84 | 0.025059666 | 2.51 |
| ControlICI | A10 | 45 | 0.02948886 | 2.95 |
| Melanoma | A10 | 411 | 0.040733399 | 4.07 |
| MelanomaICI | A10 | 143 | 0.029159869 | 2.92 |
| Control | A11 | 76 | 0.022673031 | 2.27 |
| ControlICI | A11 | 30 | 0.01965924 | 1.97 |
| Melanoma | A11 | 292 | 0.028939544 | 2.89 |
| MelanomaICI | A11 | 155 | 0.031606852 | 3.16 |
| Control | A12 | 232 | 0.069212411 | 6.92 |
| ControlICI | A12 | 29 | 0.019003932 | 1.9 |
| Melanoma | A12 | 48 | 0.004757185 | 0.48 |
| MelanomaICI | A12 | 22 | 0.004486134 | 0.45 |
| Control | A13 | 40 | 0.011933174 | 1.19 |
| ControlICI | A13 | 26 | 0.017038008 | 1.7 |
| Melanoma | A13 | 87 | 0.008622398 | 0.86 |
| MelanomaICI | A13 | 40 | 0.008156607 | 0.82 |
| Control | A14 | 21 | 0.006264916 | 0.63 |
| ControlICI | A14 | 16 | 0.010484928 | 1.05 |
| Melanoma | A14 | 48 | 0.004757185 | 0.48 |
| MelanomaICI | A14 | 33 | 0.006729201 | 0.67 |
| Control | A15 | 17 | 0.005071599 | 0.51 |
| ControlICI | A15 | 6 | 0.003931848 | 0.39 |
| Melanoma | A15 | 33 | 0.003270565 | 0.33 |
| MelanomaICI | A15 | 28 | 0.005709625 | 0.57 |
| Control | A16 | 11 | 0.003281623 | 0.33 |
| ControlICI | A16 | 2 | 0.001310616 | 0.13 |
| Melanoma | A16 | 37 | 0.003666997 | 0.37 |
| **Dataset** | **Astrocyte_cluster** | **cell_count** | **proportion** | **% proportion** |
| MelanomaICI | A16 | 31 | 0.00632137 | 0.63 |
| Control | A17 | 6 | 0.001789976 | 0.18 |
| ControlICI | A17 | 8 | 0.005242464 | 0.52 |
| Melanoma | A17 | 30 | 0.002973241 | 0.3 |
| MelanomaICI | A17 | 19 | 0.003874388 | 0.39 |
| Control | A18 | 5 | 0.001491647 | 0.15 |
| ControlICI | A18 | 2 | 0.001310616 | 0.13 |
| Melanoma | A18 | 22 | 0.002180377 | 0.22 |
| MelanomaICI | A18 | 13 | 0.002650897 | 0.27 |

**Supplemental Table 7. Relative distribution of Astrocyte subcluster across the datasets.**

Raw values corresponding to the stacked bar plots showing the relative proportions of Astrocyte subclusters across the datasets. For each dataset, cells assigned to each cluster were counted and normalized to the total microglial cell number. Pairwise difference in cluster distribution between selected groups were evaluated using Fischer’s exact test, followed by Benjamini-Hochberg correction for multiple comparison. Significance level: adjusted P<0.05

**Suppl. Table 8**

| **Dataset** | **Oligo_cluster** | **cell_count** | **proportion** | **% proportion** |
| --- | --- | --- | --- | --- |
| Control | O1 | 703 | 0.115227012 | 11.52 |
| ControlICI | O1 | 153 | 0.043965517 | 4.4 |
| Melanoma | O1 | 2769 | 0.187690639 | 18.77 |
| MelanomaICI | O1 | 1542 | 0.202282566 | 20.23 |
| Control | O2 | 844 | 0.138337977 | 13.83 |
| ControlICI | O2 | 589 | 0.169252874 | 16.93 |
| Melanoma | O2 | 2116 | 0.143428455 | 14.34 |
| MelanomaICI | O2 | 875 | 0.114784206 | 11.48 |
| Control | O3 | 1135 | 0.186035076 | 18.6 |
| ControlICI | O3 | 707 | 0.20316092 | 20.32 |
| Melanoma | O3 | 1124 | 0.076187894 | 7.62 |
| MelanomaICI | O3 | 903 | 0.1184573 | 11.85 |
| Control | O4 | 920 | 0.150794952 | 15.08 |
| ControlICI | O4 | 708 | 0.203448276 | 20.34 |
| Melanoma | O4 | 1107 | 0.075035586 | 7.5 |
| MelanomaICI | O4 | 826 | 0.10835629 | 10.84 |
| Control | O5 | 564 | 0.092443862 | 9.24 |
| ControlICI | O5 | 198 | 0.056896552 | 5.69 |
| Melanoma | O5 | 1693 | 0.114756321 | 11.48 |
| MelanomaICI | O5 | 834 | 0.109405746 | 10.94 |
| Control | O6 | 518 | 0.084904114 | 8.49 |
| ControlICI | O6 | 252 | 0.072413793 | 7.24 |
| Melanoma | O6 | 1232 | 0.083508439 | 8.35 |
| MelanomaICI | O6 | 608 | 0.079758625 | 7.98 |
| Control | O7 | 358 | 0.058678905 | 5.87 |
| ControlICI | O7 | 159 | 0.045689655 | 4.57 |
| Melanoma | O7 | 992 | 0.067240561 | 6.72 |
| MelanomaICI | O7 | 601 | 0.078840352 | 7.88 |
| Control | O8 | 350 | 0.057367645 | 5.74 |
| **Dataset** | **Oligo_cluster** | **cell_count** | **proportion** | **% proportion** |
| ControlICI | O8 | 144 | 0.04137931 | 4.14 |
| Melanoma | O8 | 597 | 0.040466346 | 4.05 |
| MelanomaICI | O8 | 412 | 0.054046963 | 5.4 |
| Control | O9 | 169 | 0.027700377 | 2.77 |
| ControlICI | O9 | 166 | 0.047701149 | 4.77 |
| Melanoma | O9 | 648 | 0.04392327 | 4.39 |
| MelanomaICI | O9 | 361 | 0.047356684 | 4.74 |
| Control | O10 | 36 | 0.005900672 | 0.59 |
| ControlICI | O10 | 7 | 0.002011494 | 0.2 |
| Melanoma | O10 | 715 | 0.048464719 | 4.85 |
| MelanomaICI | O10 | 63 | 0.008264463 | 0.83 |
| Control | O11 | 175 | 0.028683822 | 2.87 |
| ControlICI | O11 | 159 | 0.045689655 | 4.57 |
| Melanoma | O11 | 268 | 0.018165797 | 1.82 |
| MelanomaICI | O11 | 165 | 0.021645022 | 2.16 |
| Control | O12 | 114 | 0.018685461 | 1.87 |
| ControlICI | O12 | 74 | 0.021264368 | 2.13 |
| Melanoma | O12 | 286 | 0.019385888 | 1.94 |
| MelanomaICI | O12 | 169 | 0.022169749 | 2.22 |
| Control | O13 | 41 | 0.00672021 | 0.67 |
| ControlICI | O13 | 32 | 0.009195402 | 0.92 |
| Melanoma | O13 | 417 | 0.028265438 | 2.83 |
| MelanomaICI | O13 | 85 | 0.011150466 | 1.12 |
| Control | O14 | 25 | 0.004097689 | 0.41 |
| ControlICI | O14 | 16 | 0.004597701 | 0.46 |
| Melanoma | O14 | 497 | 0.033688063 | 3.37 |
| MelanomaICI | O14 | 12 | 0.001574183 | 0.16 |
| Control | O15 | 62 | 0.010162268 | 1.02 |
| ControlICI | O15 | 58 | 0.016666667 | 1.67 |
| Melanoma | O15 | 85 | 0.00576154 | 0.58 |
| MelanomaICI | O15 | 71 | 0.009313918 | 0.93 |
| Control | O16 | 49 | 0.00803147 | 0.8 |
| **Dataset** | **Oligo_cluster** | **cell_count** | **proportion** | **% proportion** |
| ControlICI | O16 | 18 | 0.005172414 | 0.52 |
| Melanoma | O16 | 121 | 0.008201722 | 0.82 |
| MelanomaICI | O16 | 70 | 0.009182736 | 0.92 |
| Control | O17 | 37 | 0.00606458 | 0.61 |
| ControlICI | O17 | 39 | 0.011206897 | 1.12 |
| Melanoma | O17 | 40 | 0.002711313 | 0.27 |
| MelanomaICI | O17 | 26 | 0.003410731 | 0.34 |
| Control | O18 | 1 | 0.000163908 | 0.02 |
| ControlICI | O18 | 1 | 0.000287356 | 0.03 |
| Melanoma | O18 | 46 | 0.00311801 | 0.31 |
| MelanomaICI | O18 | 0 | 0 | 0 |

**Supplemental Table 8. Relative distribution of Oligodendrocytes subcluster across the datasets.**

Raw values corresponding to the stacked bar plots showing the relative proportions of Oligodendrocytes subclusters across the datasets. For each dataset, cells assigned to each cluster were counted and normalized to the total microglial cell number. Pairwise difference in cluster distribution between selected groups were evaluated using Fischer’s exact test, followed by Benjamini-Hochberg correction for multiple comparison. Significance level: adjusted P<0.05
